# A multi‐centre, prospective trial of a methylation‐based liquid biopsy for early detection of liver cancer in high‐risk populations

**DOI:** 10.1002/ctm2.70687

**Published:** 2026-05-18

**Authors:** Ruohan Zhang, Xinrong Yang, Guangming Li, Yinan Deng, Jibing Liu, Hongjun Gao, Jie Zhao, Jianwen Cheng, Xiaofei Zhao, Yang Yang, Zhen Wu, Shuangzhen Gu, Yang Wu, Zhongying Ma, Yanli Liu, Yan Kang, Guangpeng Zhou, Hua Li, Yonghong Zhang, Xiaoliang Han, Jia Fan, Jian Zhou, Kefeng Dou, Kaishan Tao

**Affiliations:** ^1^ Department of Hepatobiliary Surgery Xijing Hospital Air Force Medical University Xi'an China; ^2^ Department of Liver Surgery & Transplantation Liver Cancer Institute Zhongshan Hospital Fudan Universi ty and Key Laboratory of Carcinogenesis and Cancer Invasion Ministry of Education Shanghai China; ^3^ Department of General Surgery Center Beijing Youan Hospital Capital Medical University Beijing China; ^4^ Clinical Center for Liver Cancer Capital Medical University Beijing China; ^5^ Department of Hepatic Surgery & Liver Transplantation The Third Affiliated Hospital of Sun Yat‐sen University Guangzhou China; ^6^ Department of Interventional Therapy I Shandong Cancer Hospital and Institute Shandong First Medical University and Shandong Academy of Medical Sciences Jinan Shandong China; ^7^ Department of Clinical Laboratory State Key Laboratory of Molecular Oncology National Cancer Center/National Clinical Research Center for Cancer/Cancer Hospital Chinese Academy of Medical Sciences and Peking Union Medical College, Chaoyang District Beijing China; ^8^ Department of Clinical Laboratory Shanxi Province Cancer Hospital Shanxi Hospital Chinese Academy of Medical Sciences Taiyuan Shanxi China; ^9^ Department of Research & Development BioChain (Beijing) Science & Technology, Inc. Beijing China; ^10^ Department of Pharmacy Xijing Hospital Air Force Medical University Xi'an China; ^11^ Shandong Provincial Key Laboratory of Precision Oncology Shandong Cancer Hospital and Institute Shandong First Medical University and Shandong Academy of Medical Sciences Jinan Shandong China; ^12^ Interventional therapy Center for liver diseases and cancer Beijing Youan Hospital Capital Medical University Beijing China

**Keywords:** cfDNA, early detection, liquid biopsy, liver cancer diagnosis, methylation marker, non‐invasive diagnosis

## Abstract

**Background and aims:**

Existing imaging and serum‐marker assays miss many early liver cancers, especially in high‐risk chronic liver disease carriers. We aimed to create a highly accurate, non‐invasive, methylation‐based liquid biopsy for early detection.

**Methods:**

We used a comprehensive, multi‐platform, multi‐cohort strategy for marker discovery, starting with methylation profiling of hepatocellular carcinoma samples from TCGA and in‐house cohorts. From 30 initial candidates, nine highly liver‐specific methylation markers were shortlisted, and three optimal cfDNA markers (RNF135, CHFR, PAX5) were selected to develop a robust diagnostic model, tuned in a training set (*N* = 280) and locked in an internal testing set (*N* = 124). The model was then validated in a prospective, large‐scale trial conducted at four geographically distinct Chinese centres.

**Results:**

The clinical trial included 1097 participants from two groups, (i) a diagnosing group (*N* = 646) that prospectively enrolled individuals without prior diagnostic results and represented a real‐world high‐risk population, and (ii) a diagnosed group recruited after pathology confirmation. Overall, the model achieved 94.43% (95% confidence interval, 92.12–96.09%) sensitivity and 95.16% (92.78–96.78%) specificity for liver cancer, with stage‐I sensitivity of 93.10% (89.78–95.40%). Within the diagnosing group, overall sensitivity was 93.99% (91.28–95.90%), and for the 267 stage‐I cases, it reached 92.88% (89.15–95.39%). As for specificity, it remained high across confounders: 92.78% (85.84–96.46%) in cirrhosis, 91.74% (85.46–95.45%) in other‐cancer interference samples. Besides, the model outperformed the traditional liver cancer biomarker AFP and showed changes in methylation signals before and after surgery, suggesting a possible role in perioperative monitoring. Each centre independently reported sensitivities and specificities exceeding 90%, demonstrating robust geographic performance.

**Conclusions:**

Using a systematic marker‐discovery pipeline and a multi‐centre prospective cohort, we developed a methylation‐based liquid biopsy that reliably detects early liver cancer in high‐risk populations.

**Clinical trial number:**

Chictr.org identifier: ChiCTR2400092883.

**Key points:**

- Three cfDNA methylation markers, RNF135, CHFR and PAX5, were identified for liver cancer liquid biopsy.
- A three‐marker diagnostic model based on qMSP was established for highly accurate non‐invasive detection of liver cancer.
- The LC‐HMC model achieved 94.43% sensitivity and 95.16% specificity in the clinical trial.
- The model detected stage‐I liver cancer with a sensitivity of 93.10%.

## Graphical abstract

Three hypermethylated cfDNA markers specific to liver cancer, RNF135, CHFR and PAX5, were identified in peripheral blood and used to construct a diagnostic model. The model achieved 94.43% sensitivity and 95.16% specificity in a large clinical validation cohort, with a sensitivity of 93.1% for stage‐I liver cancer.

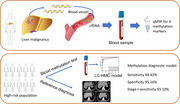

## 1 INTRODUCTION

Primary liver cancer is a major global malignancy, with over 865 000 new cases and 757 000 deaths in 2022, ranking sixth in incidence and third in cancer‐related mortality worldwide.^1^ Hepatitis B virus (HBV) and aflatoxin are key etiological factors, while hepatitis C virus (HCV), genetic susceptibility, alcoholism and fatty liver diseases also contribute.^2^, ^3^, ^4^ Due to its insidious onset and limited treatment options, liver cancer has a poor prognosis in less developed regions, with late diagnosis being a major contributing factor, underscoring the importance of prevention and early detection.^5^, ^6^

Early screening is critical for improving outcomes. Current methods rely on medical imaging techniques like ultrasound or protein tumour markers such as alpha‐fetoprotein (AFP). However, there is currently insufficient evidence to support or refute whether AFP, ultrasound or both is adequate for the early detection of liver cancer. More well‐designed randomised trials are needed to compare the efficacy of these methods for early liver cancer screening.^7^

Recent research has focused on numerous methylation markers for early cancer diagnosis, showing promising results. Alterations in DNA methylation are common in liver cancer and may play a crucial role in its pathogenesis and diagnosis.^8^, ^9^ Hypermethylation of promoter regions in tumour suppressor genes can lead to their silencing, thereby contributing to cancer development. Furthermore, DNA methylation is relatively stable over time, allowing for non‐invasive detection using blood samples. Thus, plasma DNA methylation detection holds great potential as an early diagnostic method for liver cancer.^10^ Guo et al. conducted a multi‐centre study compared ctDNA mutation‐ and methylation‐based assays for HCC detection in a large‐scaled cohort. The authors developed and optimised a simple two‐marker qMSP assay (HCCtect) that showed a sensitivity of 78.4% and a specificity of 93.0%, including for early‐stage HCC and discrimination from high‐risk non‐malignant liver disease.^11^ Xu et al. evaluated a plasma dual‑target methylation (PDTM) qMSP test for primary liver cancer, showing high sensitivity (92.3%) and specificity (93.4%), including early‐stage disease, with methylation results highly consistent with Sanger sequencing.^12^ Moreover, another study by Guo et al. demonstrated that using 20 methylation markers in the HepaAiQ assay also yielded comparable performance for hepatocellular carcinoma (HCC) detection in a validation cohort.^13^ Besides, Kisiel et al. employed the targeted enrichment long probe quantitative amplified signal method and determined that the optimal fit for diagnosing liver cancer using six methylated DNA markers had an AUC of 0.96, a sensitivity of 95% and a specificity of 92%.^14^ These findings underscored the potential of DNA methylation biomarkers in improving early detection and diagnosis of liver cancer.

In this study, we identified candidate hypermethylated DNA regions through integrated analysis of public datasets and in‐house clinical samples. Using qMSP, we developed a detection method for three selected DNA methylation markers and trained the LC‐HMC diagnostic model. The model's performance was validated in a clinical trial with 1097 participants (521 liver cancer patients, 455 controls and 121 other cancer patients), confirming the efficacy of this non‐invasive approach for liver cancer diagnosis and early detection.

## 2 MATERIALS AND METHODS

### 2.1 Study design

**FIGURE 1:**
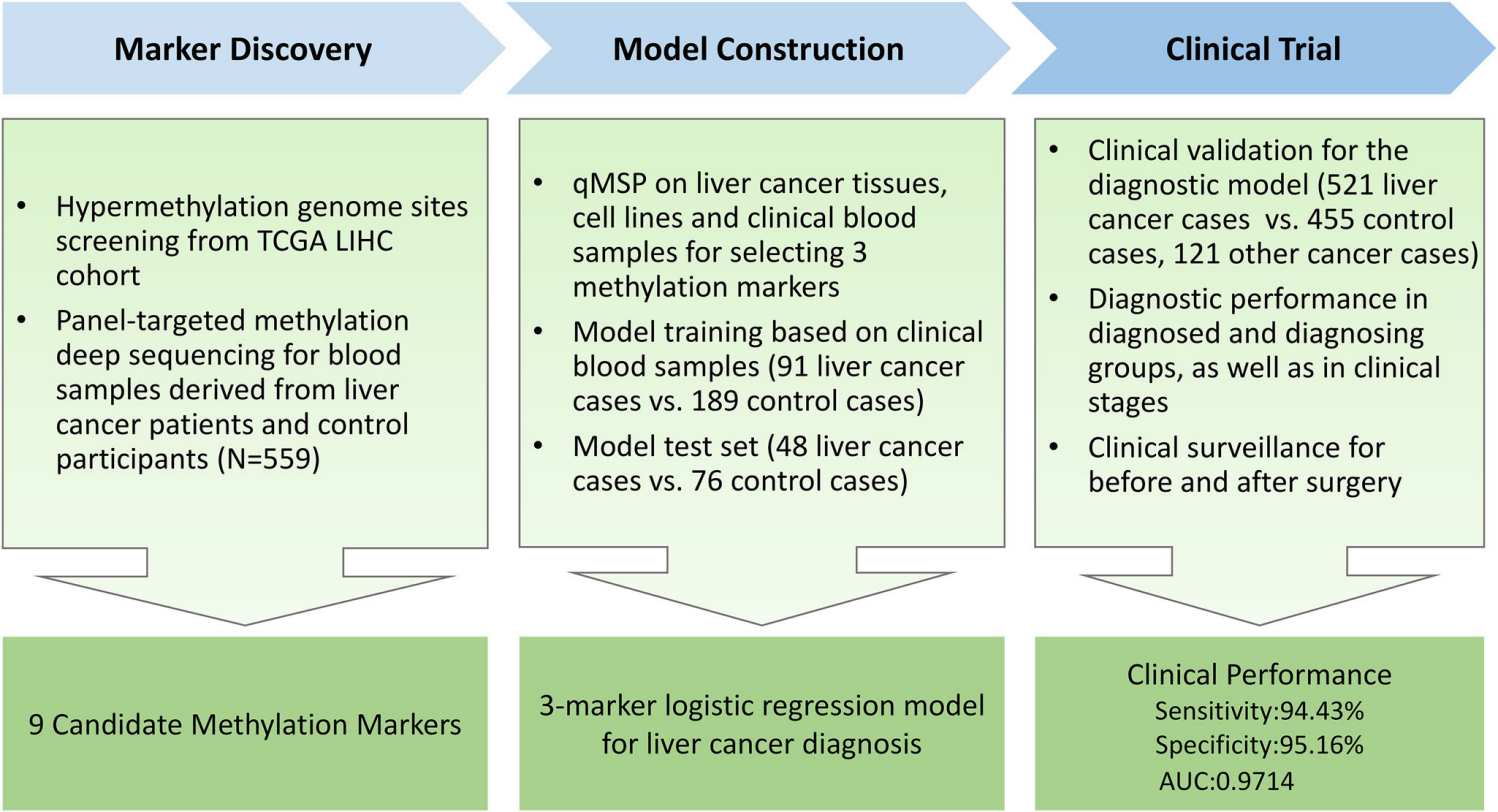
Workflow of the study. The workflow indicating three stages of the study, including marker discovery, model construction and clinical trial. *Abbreviations*: qMSP, quantitative methylation specific PCR; LIHC, liver hepatocellular carcinoma.

This study comprised three primary phases (Figure 1). (1) *Marker Discovery Phase*: Public data from the TCGA LIHC DNA methylation 450K array dataset were used to analyse methylation differences in liver cancer tissues. The public data were downloaded from the data portal (https://portal.gdc.cancer.gov/projects/TCGA‐LIHC), including 377 liver cancer tissues versus 50 adjacent normal tissues. Among the differentially methylated genes, hotspot genes associated with liver cancer, as identified in the previous investigations, were compiled into a methylation‐detecting panel. Thus, a deep methylation sequencing panel was constructed, which was applied to 559 clinical plasma samples from three centres to identify liver cancer‐specific hypermethylation genomic regions, termed candidate methylation markers. (2) *Model Construction Phase*: Candidate methylation markers were further tested. Primers and probes were designed for them. And qMSP was performed on various sample types, including 24 liver cancer cell lines, 32 liver cancer tissue samples and 41 blood samples (32 from healthy individuals and nine from liver cancer patients). The diagnostic ability of each candidate was evaluated, leading to the selection of three methylation markers. Subsequentially, the qMSP experiment focusing on these three markers was conducted on 404 clinical blood samples, which were divided into training and testing sets. A logistic regression model based on the three methylation markers was then developed to diagnose liver cancer. (3) *Clinical Trial Phase*: This phase involved a multi‐centre clinical trial to verify the performance of the LC‐hypermethylated markers‐combined (LC‐HMC) model. The trial recruited 1097 clinical participants from four centres to evaluate the LC‐HMC model's sensitivity, specificity and other relevant metrics.

### 2.2 Ethics

All the procedures of the study were conducted in accordance with the Helsinki Declaration of 1964 and later versions. All the participants in the study provided written informed consent and were duly informed about the utilisation of plasma and test outcomes. The study was approved by the local ethics committees of each hospital involved.

The ethical numbers are as follows.

#### 2.2.1 Centres participated in preclinical investigation

The centres that participated in the preclinical investigations were: Cancer Hospital of Shandong First Medical University, SDTHEC 2023011011; The First Affiliated Hospital of Fourth Military Medical University, Xijing Hospital, QX20231022‐1; Shanxi Cancer Hospital, KY2023103.

#### 2.2.2 Centres participated in clinical trial

The centres that participated in the clinical trial were: the First Affiliated Hospital of Fourth Military Medical University, Xijing Hospital, QX20231022‐C‐1; Zhongshan Hospital, Fudan University, 2023–077; Beijing Youan Hospital, Capital Medical University, LL‐2023‐075‐S; The Third Affiliated Hospital, Sun Yat‐sen University, SJ2023‐004‐01.

### 2.3 Preclinical investigation sample collection

Preclinical samples were provided by three centres, Cancer Hospital of Shandong First Medical University, the First Affiliated Hospital of Fourth Military Medical University and Shanxi Cancer hospital. Ethical approval numbers are mentioned in the *Ethics Statement* section.

The 559 clinical blood samples, which involved in marker discovery phase, included an initial batch of 93 samples (30 healthy individuals, 31 liver cancer patients and 32 patients with other cancers) from the Cancer Hospital of Shandong First Medical University. The second batch included 466 samples (67 cases of colorectal cancer, 30 cases of oesophageal cancer, 31 cases of liver cancer, 51 cases of lung cancer, 37 cases of ovarian cancer, 46 cases of gastric cancer, 26 cases of thyroid cancer and 178 healthy individuals) from the other two centres.

The samples involved in model construction phase were obtained from the three centres. There were 32 liver cancer tissue samples, 32 healthy blood samples and nine liver cancer patient blood samples being used to select the three methylation markers. And there were 404 blood samples for model development. From these, 280 samples were allocated to the training set (91 liver cancer patients and 189 controls, controls included 128 benign disease cases and 61 healthy individuals), and 124 samples were allocated to the testing set (48 liver cancer patients and 76 controls, controls included 35 benign disease cases and 41 healthy individuals).

### 2.4 Clinical trial design and participants

The clinical trial (trial registration number: ChiCTR2400092883) was designed as a multi‐centre, parallel, blind‐label clinical test, with a total of 1097 participants. The participants were recruited from four centres, the First Affiliated Hospital of Fourth Military Medical University, Zhongshan Hospital Fudan University, Beijing Youan Hospital and the Third Affiliated Hospital of Sun Yat‐sen University. The trial included patients with primary liver cancer (case group), benign liver disease (control group) and other types of cancer (interference group, comprising five types of non‑hepatic primary malignancies).

#### 2.4.1 Sample size estimation

The required sample size was estimated based on achieving a 95% confidence interval with a precision of ±5% around an expected sensitivity/specificity of 90%, according to the formula

$$n=Z_{\left(1-\frac{\alpha }{2}\right)}^{2}\frac{P\left(1-P\right)}{d^{2}}$$

This yielded a minimum of 139 liver cancer cases and 139 benign disease cases. However, to ensure adequate statistical power for pre‐specified key subgroup analyses (e.g., stage‐I liver cancer and benign liver tumours), the total sample size was prospectively expanded. As these subgroups were expected to comprise only a proportion of the overall cohort (e.g., stage‐I liver cancer was pre‐estimated to account for 25–35% of all liver cancer cases, requiring a total liver cancer enrolment of at least 139/30% = 464 cases to obtain 139 stage‐I cases), a larger overall enrolment was required to ensure sufficient sample size within key subgroup. Based on this consideration, the planned sample size was set at approximately 1000 participants, with a liver cancer to benign liver disease ratio of roughly 1:1, while also accounting for an anticipated ∼10% rate of unqualified clinical samples.

#### 2.4.2 Inclusion and exclusion of participants

Given that the intended application of this liver cancer methylation diagnostic model is in (1) patients at high risk for liver cancer and (2) patients presenting to clinics with liver‑related symptoms for initial diagnosis or auxiliary diagnosis of liver cancer, the clinical trial was designed with two parallel enrolment pathways: a diagnosed group and a diagnosing group. The diagnosed group includes participants who already had a confirmed diagnosis based on the clinical reference standard at the time of enrolment. This group was used to verify that the methylation‑based diagnostic model can distinguish liver cancer from benign liver diseases and specifically identify primary liver cancer among different cancer types. The diagnosing group includes participants whose liver cancer status was unknown at the time of blood sample collection, that is, the investigators were blinded to the reference standard diagnosis at enrolment. This group was designed to simulate real‑world clinical scenarios and to evaluate whether the methylation diagnostic model can be applied for early diagnosis of liver cancer in high‑risk populations.

#### 2.4.3 Diagnosed group inclusion criteria

1. Liver cancer
  Patients with primary liver cancer confirmed by reference methods (dynamic contrast‑enhanced computed tomography (CT)/magnetic resonance imaging (MRI) or liver biopsy). Blood samples for methylation biomarker testing were collected after the diagnosis was established and before initiation of any anti‑cancer treatment.
2. Benign disease
  Patients with a confirmed benign liver disease, including hepatitis, benign liver tumours, liver cirrhosis and fatty liver disease. Participants were required to be confirmed as free of liver cancer and any other malignancy, based on comprehensive clinical evaluation by specialists.
3. Other cancers
  Patients with confirmed other gastrointestinal malignancies, diagnosed by appropriate reference methods (CT, MRI, endoscopy, biopsy), including extrahepatic cholangiocarcinoma, oesophageal cancer, gastric cancer, colorectal cancer and pancreatic cancer. These patients were required to be confirmed as free of primary liver cancer. Blood samples for methylation biomarker testing were collected before any anti‑cancer treatment.

#### 2.4.4 Diagnosing group inclusion criteria

1. Liver cancer
  (a) Symptomatic patients with suspected liver lesions. Patients presenting with liver‑related or non‑specific symptoms (such as right upper abdominal pain, gastrointestinal symptoms including bloating, loss of appetite, nausea, vomiting, diarrhoea, upper abdominal mass, fever, fatigue or weight loss) who underwent ultrasound examination and were found to have a suspected liver space‑occupying lesion. These patients were enrolled and had blood samples collected for methylation biomarker testing before the reference standard examination. They then underwent reference standard evaluation (dynamic contrast‑enhanced CT/MRI, or pathological diagnosis). Patients subsequently confirmed to have primary liver cancer were included in the liver cancer group of the diagnosing group.
  (b) Guideline‑defined high‑risk population for HCC, including individuals with chronic HBV infection, chronic HCV infection, long‑term heavy alcohol consumption, fatty liver disease, history of consuming food contaminated with aflatoxin, liver cirrhosis and family history of liver cancer. These individuals underwent ultrasound examination and blood testing for methylation biomarkers. Those with liver space‑occupying lesions detected by ultrasound underwent further reference standard evaluation. Participants subsequently confirmed to have primary liver cancer were included.
2. Benign disease
  (a) Symptomatic patients described above who had liver space‑occupying lesions on ultrasound but were subsequently confirmed by reference standard examinations (dynamic contrast‑enhanced CT/MRI or pathological diagnosis) to have benign liver lesions were enrolled into the benign disease group of the diagnosing group.
  (b) Symptomatic patients described above who had no liver space‑occupying lesion on ultrasound. These patients underwent blood sampling for methylation biomarker testing and other necessary examinations. Clinicians performed a comprehensive evaluation, including lifestyle, medical history, serum biomarkers, viral hepatitis test results and imaging findings, to confirm the diagnosis of benign liver disease and to exclude any malignancy. Such patients were enrolled into the benign disease group of the diagnosing group.
  (c) Guideline‑defined high‑risk population for HCC described above who had liver space‑occupying lesions on ultrasound but were subsequently confirmed by reference standard examinations to have benign liver lesions were enrolled into the benign disease group.
  (d) Guideline‑defined high‑risk population for HCC with no liver space‑occupying lesion detected on ultrasound. These participants underwent blood sampling for methylation biomarker testing and other examinations. As in criterion (2b), clinicians performed a comprehensive evaluation (lifestyle, disease history, serum biomarkers, viral hepatitis status and imaging findings). Those confirmed to have benign liver disease and to be free of malignancy were enrolled.
3. Other cancers
  Potential patients with suspected other gastrointestinal malignancies who were scheduled to undergo reference standard examinations (CT, MRI, endoscopy and biopsy) were collected blood samples for methylation biomarker testing before the diagnosis was known. Participants subsequently confirmed to have one of the following malignancies: extrahepatic cholangiocarcinoma, oesophageal cancer, gastric cancer, colon cancer, pancreatic cancer and confirmed to be free of liver cancer were enrolled into the other cancer group.

#### 2.4.5 Perioperative surveillance

Participants included in perioperative surveillance were a subset of the diagnosing group who met the liver cancer inclusion criteria described above. Those liver cancer patients who underwent curative resection at the participating centre and from whom a postoperative blood sample was successfully collected were included in the perioperative surveillance cohort. After enrolment and before any anti‑cancer treatment, baseline blood samples were collected for methylation biomarker testing. Following tumour removal by surgery or ablation, post‑treatment blood samples were collected again for methylation biomarker testing.

#### 2.4.6 Exclusion criteria

Participants were excluded if:

- They had received any anti‑cancer treatment prior to blood sample collection for methylation analysis, except for the second blood draw in perioperative surveillance participants (post‑treatment samples).
- They had concurrent primary liver cancer and a non‑liver primary malignancy.^15^

#### 2.4.7 Methylation detection

The qMSP for RNF135, CHFR and PAX5 was performed on blood samples from the participants of clinical trial and Ct values were calculated. To maximise the input volume of the limited cfDNA template per reaction, thereby preventing low‐abundance targets from falling below the limit of detection, each qMSP assay was carried out as a single PCR reaction per sample, and duplicate holes were not included. Applying the LC‐HMC model and predefined discriminant criteria, we analysed the results of blood methylation detection. All blood methylation testing in the diagnosing group was performed in a blinded manner. The reference standard diagnostic results were unblinded for comparison and analysis only after completion of the model‐based interpretation. The study also evaluated conventional liver cancer protein marker AFP in the collected blood samples. The cutoff values of canonical biomarkers tests in four centres are displayed in Table S1.

#### 2.4.8 Primary/secondary outcomes

*Primary outcomes*: The LC‐HMC model's clinical performance was assessed in terms of overall sensitivity and specificity in liver cancer (521 liver cancer cases) and control samples (455 benign liver disease cases).

*Secondary outcomes*: Stage‐specific sensitivities, specificities in each type of benign liver diseases, specificity in other cancers and its utility in clinical surveillance before and after surgery, the sensitivity and specificity of diagnosed group and diagnosing group, respectively.

### 2.5 Statistical analysis

In this research, categorical variables were described using frequencies and percentage compositions, while quantitative variables were characterised by metrics such as the mean, standard deviation and median. To assess diagnostic effectiveness, sensitivity was defined as the ratio of accurately identified positive liver cancer cases to the total number of positive liver cancer participants included. Specificity was defined as the ratio of correctly identified negative cases to all normal individuals or those with benign diseases or other cancers. Receiver operating characteristic (ROC) curves were created using GraphPad Prism software (version 10.1.2), and the area under the ROC curve (AUC) was evaluated. To compare the differences in Ct values across multiple groups, pairwise group differences were assessed using the Wilcoxon rank‑sum test, and *p* values were adjusted for multiple testing using the Bonferroni correction. Statistical significance was defined as *p* < .05. The consistency between the methylation diagnostic model and the reference method was assessed with Kappa test.

The specific methods for data analysis and experiments are presented in Supporting Information, Methods. The methods or software used for bioinformatics data processing are referenced 16–18 in the References.^16^, ^17^, ^18^

## 3 RESULTS

### 3.1 Flowchart of the study

In this study, we followed three primary steps to identify and validate methylation markers (Figure 1). First, analysis of high‐throughput sequencing data identified nine candidate methylation markers. Second, qMSP evaluation across various sample types narrowed them to three optimal markers. Based on three markers, we developed a logistic regression model, termed the LC‐HMC model. Third, during the clinical validation phase, we conducted a large‐scale, multi‐centre, prospective and registered trial to evaluate the clinical performance of methylation markers and the LC‐HMC model. Workflow details are provided in *Materials and Methods* section.

### 3.2 Candidate methylation markers identified through genome‐wide methylation analysis

**FIGURE 2:**
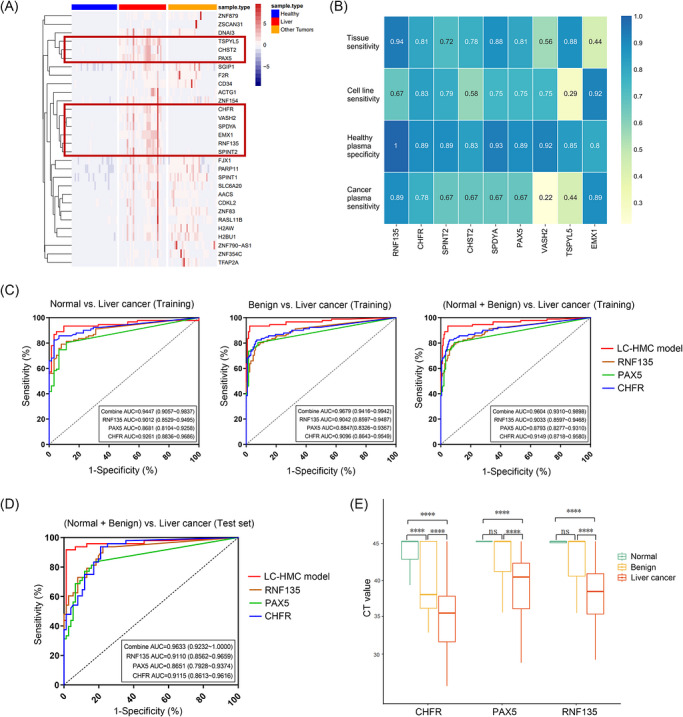
Screening of methylation markers in preclinical investigation. (A) Heatmap indicates the top 30 liver cancer hypermethylated genes based on analysing 93 samples derived from 30 healthy individuals, 31 liver cancer patients and 32 other cancers patients. The samples were collected from one centre. Color bar indicates the scaled methylation levels. Red lines encircle the nine genes which were hypermethylated in liver cancer, but not in healthy controls and other cancers. (B) The detailed numbers of performance index in each kind of samples: sensitivities of nine candidate markers when identifying 32 liver cancer tissues, sensitivities for identifying 24 liver cancer cell lines, specificities for identifying 32 healthy blood samples and sensitivities for identifying 9 liver cancer blood samples. (C) The ROC curves indicating the performance of the three‐gene diagnostic model and three methylation markers in the training set respectively in distinguishing 91 liver cancer cases from 61 healthy individuals (left panel), 91 liver cancer cases from 128 benign disease cases (middle panel), 91 liver cancer cases from 61 healthy individuals + 128 benign disease cases (right panel). (D) The ROC curves indicating the performance of the three‐gene diagnostic model and three methylation markers in the testing set when distinguishing 48 liver cancer cases from 35 benign disease cases + 41 healthy individuals. (E) The Ct values of CHFR, PAX5 and RNF135 in three types of cases in both training and testing sets. The box‐and‐whisker plot illustrates the interquartile range (IQR), with the line within the box denoting the median of the data and the whiskers extending from the box to the minimum and maximum values within 1.5 times the IQR. The Wilcoxon test was used for pairwise comparison.

In the marker‐discovery stage, we analysed TCGA LIHC dataset (377 liver cancer tissues, 50 adjacent normal tissues) and identified 901 differentially methylated regions exhibiting hypermethylation in liver cancer tissues, as visualised by a heatmap in Figure S1. Then, we evaluated these 901 regions along with their associated genes, considering genes related to liver cancer, and designed a panel containing 17 388 CpG islands to test the in‐house samples for further narrowing the scope of candidate markers. A batch of blood samples were collected from one centre and tested by the panel‐targeted methylation sequencing, which included 30 healthy individuals, 31 liver cancer patients and 32 other cancers patients. Differential analysis identified the top 30 hypermethylated markers in liver cancer, with nine markers showing low methylation both in healthy individuals and other cancer patients, highlighted in red (Figure 2A). To further confirm the specificity of these nine markers in distinguishing liver cancer from multiple cancer types, we evaluated their methylation levels in a larger cohort including 466 blood samples from two additional centres. Using controls with colorectal (*n* = 67), oesophageal (*n* = 30), lung (*n* = 51), ovarian (*n* = 37), gastric (*n* = 46) and thyroid (*n* = 26) cancers, as well as 178 healthy individuals, we confirmed the specific hypermethylation of 9 markers in 31 liver cancer cases (Figure S2).

Further screening aimed to identify markers from nine candidates that could distinguish liver cancer tissues, plasma and cell lines as positive, while identifying healthy plasma as negative through qMSP. Primers and probes were designed for the nine candidate methylation markers, and qMSP assays were performed on 32 liver cancer tissues, 24 liver cancer cell lines, 32 healthy plasma samples and nine liver cancer plasma samples. Sensitivities and specificities for each marker across the four sample types are shown in Figure 2B. Scores for the nine markers were calculated based on the formula stated in the Supporting Information, Methods, with RNF135, CHFR and SPDYA ranking highest. But most of the true‐positive and true‐negative samples identified by SPDYA were already captured by RNF135, indicating substantial redundancy between these two markers. In contrast, PAX5, despite its slightly lower score, detected a set of true‐positive cases that only partially overlapped with those identified by RNF135 (and CHFR), thereby providing complementary coverage. Therefore, RNF135 was selected for its higher score, and the fourth‐ranked gene, PAX5, was selected for its complementarity (Figure S3). Ultimately, RNF135, CHFR and PAX5 were identified as methylation markers for non‐invasive liver cancer diagnosis.

### 3.3 Liver cancer diagnostic model construction based on methylation levels of RNF135, CHFR and PAX5

Considering the complementarity of RNF135, CHFR and PAX5 in liver cancer diagnosis, we developed a logistic regression model (LC‐HMC model) based on these three markers. The qMSP assays were performed on 404 clinical blood samples, which were divided into a training set containing 280 samples deriving from 91 liver cancer patients and 189 controls (128 benign disease cases and 61 healthy individuals), and a testing set containing 124 samples deriving from 48 liver cancer patients and 76 controls (35 benign disease cases and 41 healthy individuals). The clinical characteristics of the participants involved in model construction are presented in Table S2. The model outperformed single‐marker diagnostics, with ROC curve analysis of training set showing the highest AUC values for the model in distinguishing liver cancer patients from healthy individuals, benign disease patients and all controls (Figure 2C). Validation on the testing set confirmed the model's superior performance, achieving a highest AUC of 0.9633 (95%CI, 0.9232–1.0000) (Figure 2D). Analysis of CT values revealed significantly increased methylation levels of the three markers in liver cancer (Figure 2E).

### 3.4 Multi‐centre clinical trial for validating the performance of liver cancer diagnostic model

**FIGURE 3:**
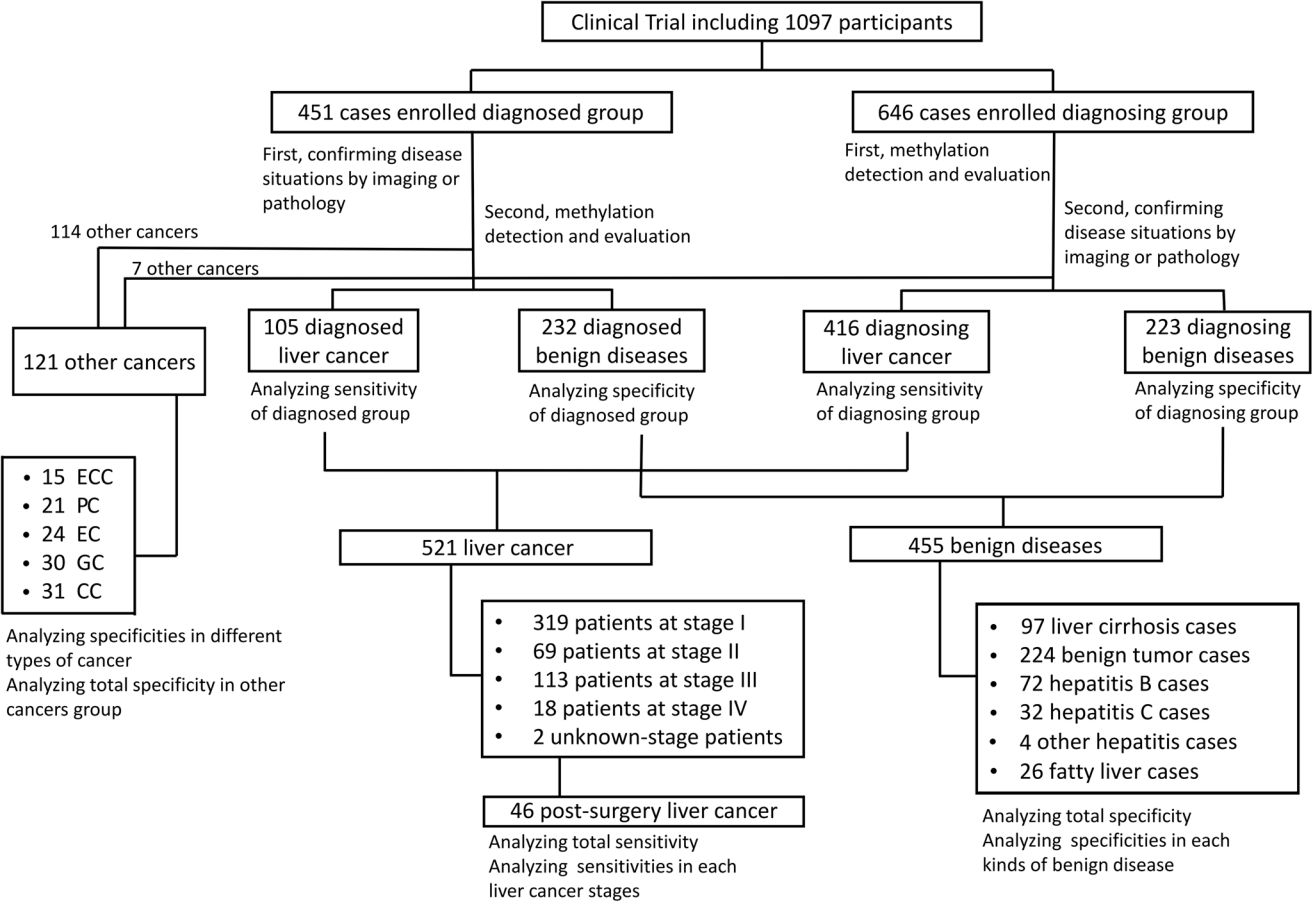
The study profile of the clinical trial. *Abbreviations*: ECC, extrahepatic cholangiocarcinoma; PC, pancreatic cancer; EC, oesophageal cancer; GC, gastric cancer; CC, colon cancer.

Since the LC‐HMC model was a promising biomarker for non‐invasive liver cancer detection, we conducted a large‐scale, multi‐centre, blind‐label clinical trial involving 1097 participants (521 liver cancer cases, 455 benign disease cases and 121 other cancer cases) from four centres. The clinical characteristics of the participants involved in the clinical trial are presented in Table S3. The work profile of the clinical trial is detailed in Figure 3. The 1097 enrolled participants were categorised into diagnosed or diagnosing groups at the time of recruitment, as detailed in *Materials and Methods* section. To ensure adequate representation of early‐stage liver cancer cases and to better approximate a real‐world early detection scenario, we intentionally enrolled a larger proportion of liver cancer patients into the diagnosing group than into the diagnosed group. Because participants in the diagnosing group had not yet received a definitive diagnosis at the time of enrolment, this strategy was used to secure a sufficient number of stage‐I liver cancer cases within this group. In contrast, most cases in the interference group were recruited from patients who had already been diagnosed with other cancers, in order to rigorously evaluate the liver cancer specificity of the diagnostic model. The final case/control ratios were 105/232 in the diagnosed group and 416/223 in the diagnosing group, and the vast majority of the 121 other cancer cases (114/121) were enrolled from the diagnosed group. Nevertheless, the distributions of key baseline characteristics, such as age and sex, were similar between the two groups. The respective clinical characteristics of the diagnosed and diagnosing group are summarised in Table S4. The two groups were analysed sensitivity and specificity respectively, followed by overall performance evaluation.

**FIGURE 4:**
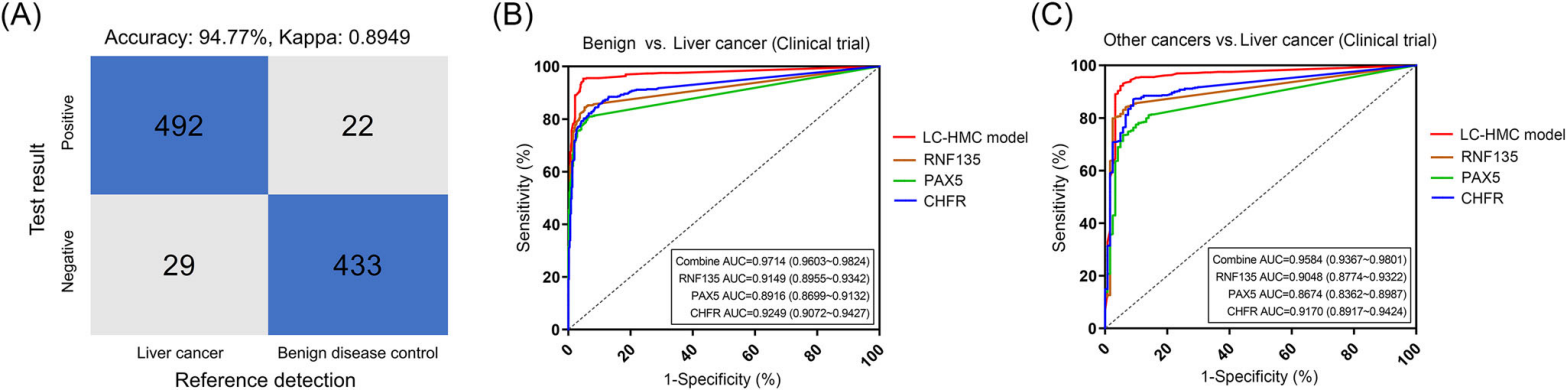
The performance of blood methylation detection in the clinical trial. (A) Confusion matrix comparing the LC‐HMC model‐observed classifications with reference detection methods in the clinical trial cohort. Liver cancer (*n* = 521) and benign disease control (*n* = 455) according to reference detection methods were performed blood methylation detection. The blue grids indicated numbers of true‐positive cases or true‐negative cases. The grey grids indicated numbers of false‐detection cases. (B) The ROC curves indicating the performance of the three‐gene diagnostic model and three methylation markers in the clinical trial cohort when distinguishing 521 liver cancer cases from 455 benign disease cases. (C) The ROC curves indicating the performance of distinguishing 521 liver cancer cases from 121 other cancers cases.

We analysed the sensitivities in 521 liver cancer cases (105 in the diagnosed group and 416 in the diagnosing group), specificities in 455 benign disease cases (232 in diagnosed group and 223 in the diagnosing group) and corresponding accuracy. The LC‐HMC model achieved an overall sensitivity of 94.43% (95%CI, 92.12–96.09%) and specificity of 95.16% (95%CI, 92.78–96.78%) (Table S5). The confusion matrix compared the diagnostic results of the LC‐HMC model and the reference detection methods, indicating an overall accuracy of 94.77% (Figure 4A). In the clinical trial cohort, the LC‐HMC model achieved an AUC of 0.9714 (95%CI, 0.9603–0.9824) in distinguishing liver cancer from benign liver diseases (Figure 4B) and an AUC of 0.9584 (95%CI, 0.9367–0.9801) in distinguishing liver cancer from other cancers (Figure 4C). Notably, the sensitivity in the diagnosing group (93.99%, 95%CI, 91.28–95.90%) was comparable to that of the diagnosed group (96.19%, 95%CI, 90.61–98.51%), and the sensitivity in the stage‐I cases (*N* = 267) of the diagnosing group reached 92.88% (95%CI, 89.15–95.39%), suggesting the LC‐HMC model's potential for liver cancer screening in high‐risk populations (Table S5).

Moreover, we investigated whether the sensitivity of the LC‐MHC model was associated with the age, cancer subtypes or tumour differentiation of liver cancer patients. Our findings revealed that the model's sensitivity exceeded 90% across various subgroups with these clinical characteristics (Table 1). The LC‐HMC model could effectively distinguished liver cancer from benign diseases, with specificities of 92.78% (95%CI, 85.84–96.46%) (cirrhosis), 95.54% (95%CI, 91.98–97.56%) (benign tumours), 95.83% (95%CI, 88.45–98.57%) (hepatitis B), 93.75% (95%CI, 79.85–98.27%) (hepatitis C), 100.00% (95%CI, 51.01–100.00%) (other hepatitis) and 100.00% (95%CI, 87.13–100.00%) (fatty liver). Using other cancers (*n* = 121) as controls, the overall specificity was 91.74% (95%CI, 85.46–95.45%), slightly lower than with benign diseases (95.16%) (95%CI, 92.78–96.78%). Among other cancers, we observed specificities of 93.33% (95%CI, 70.18–98.81%) in extrahepatic cholangiocarcinoma, 90.48% (95%CI, 71.09–97.35%) in pancreatic cancer, 91.67% (95%CI, 74.16–97.69%) in esophageal cancer, 90.00% (95%CI, 74.38–96.54%) in gastric cancer and 93.55% (95%CI, 79.28–98.21%) in colon cancer (Table 2). We also examined whether the specificity of the LC‐MHC model varied among benign disease controls or other cancers across different age groups, but the specificity consistently remained at or above 90% (Table 2). The sensitivity and specificity of the four centres are displayed in Table S6. The sensitivity and specificity in male and female showed no significant difference (Table S7).

**TABLE 1:** The sensitivity of LC‐HMC model in liver cancer patients with different clinical characteristics.

Characteristics	Case number	Positive cases	Sensitivity	95% CI
Age				
<40	31	30	96.77%	83.81–99.95%
40–59	259	246	94.98%	91.62–97.23%
60–79	223	208	93.27%	88.79–96.35%
≥80	8	8	100.00%	63.06–100.00%
Liver cancer subtypes				
Hepatocellular carcinoma	500	473	94.60%	91.49–94.88%
Mixed hepatocellular–cholangiocarcinoma	21	19	90.48%	67.76–97.85%
Tumour differentiation				
Low differentiated	35	32	91.43%	82.91–98.62%
Medium‐low differentiation	38	36	94.74%	86.03–99.42%
Medium differentiated	228	215	94.30%	89.92–95.82%
Medium‐high differentiation	11	11	100.00%	71.58–100.00%
High differentiated	14	13	92.86%	67.66–99.72%
Unknown	195	185	94.87%	88.49–96.42%

**TABLE 2:** The specificity of the LC‐HMC model across various benign diseases, other cancers and different age categories.

Characteristics	Case number	Negative cases	Specificity	95% CI
Benign disease control	455			
Liver cirrhosis	97	90	92.78%	85.84–96.46%
Liver benign tumour	224	214	95.54%	91.98–97.56%
Hepatitis B	72	69	95.83%	88.45–98.57%
Hepatitis C	32	30	93.75%	79.85–98.27%
Other hepatitis	4	4	100.00%	51.01–100.00%
Fatty liver	26	26	100.00%	87.13–100.00%
Other cancers	121			
Extrahepatic cholangiocarcinoma	15	14	93.33%	70.18–98.81%
Pancreatic cancer	21	19	90.48%	71.09–97.35%
Oesophageal cancer	24	22	91.67%	74.16–97.69%
gastric cancer	30	27	90.00%	74.38–96.54%
Colon cancer	31	29	93.55%	79.28–98.21%
Age categories				
Benign disease control				
<40	125	118	94.40%	88.89–97.26%
40–59	230	221	96.09%	92.74–97.93%
60–79	98	92	93.88%	87.28–97.17%
≥80	2	2	100.00%	34.24–100.00%
Other cancers				
<40	4	4	100.00%	51.01–100.00%
40–59	37	33	89.19%	75.29–95.72%
60–79	80	74	92.50%	84.59–96.52%

### 3.5 Superior performance of the LC‐HMC model in clinical inspection of early liver cancer

**FIGURE 5:**
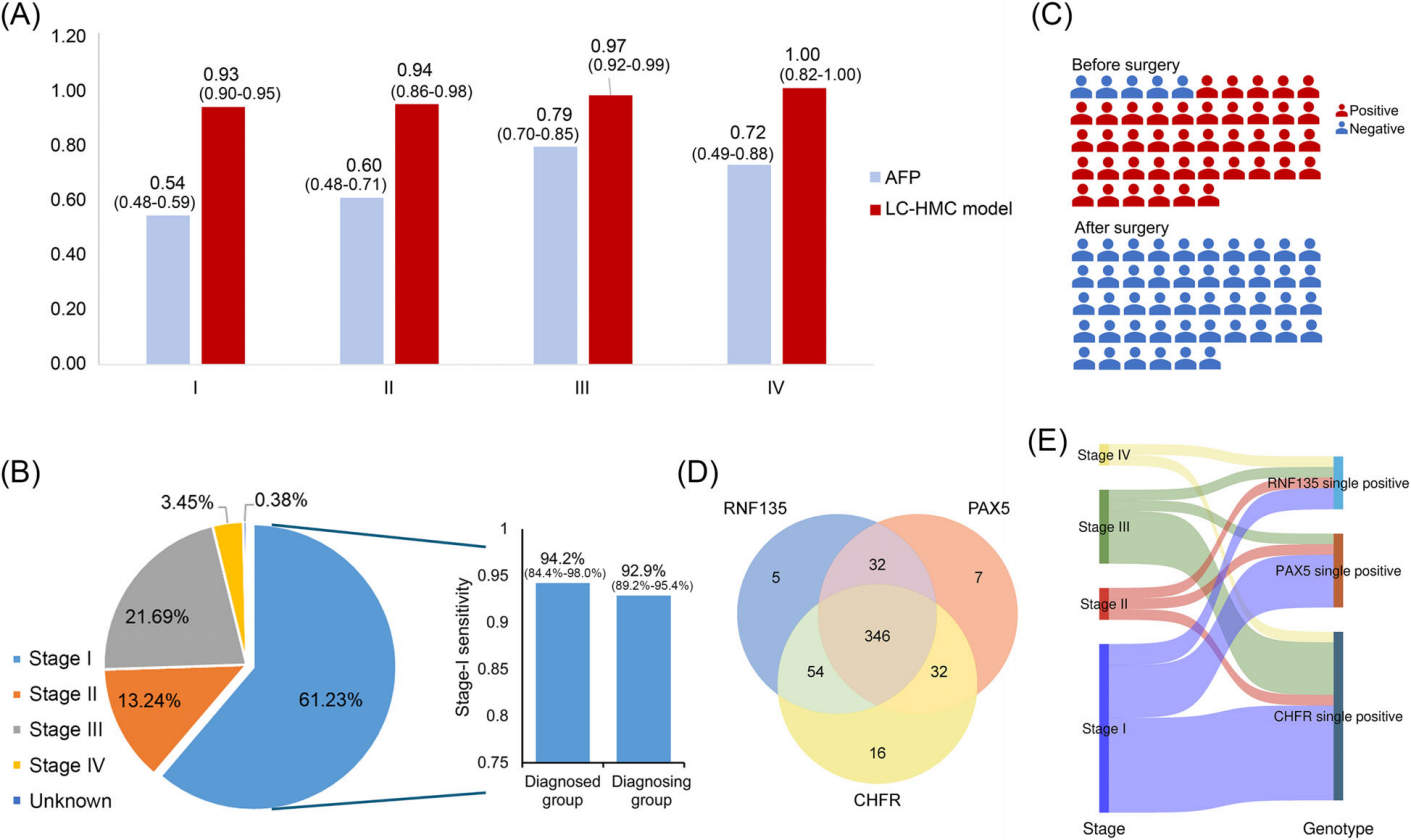
Multi‐aspects analysis of the LC‐HMC model performance in 1097 participants. (A) Sensitivities of the LC‐HMC model and the specific liver cancer protein marker, AFP, respectively in each LC stage. (B) The pie chart shows the proportions of each clinical stages in liver cancer cases. The right panel shows the stage‐I sensitivities in diagnosed group and diagnosing group. (C) The testing results of the LC‐HMC model in 46 LC patients before and after surgery. (D) Venn diagram shows numbers of true‐positive cases distinguished by each methylation marker, as well as displaying the numbers of overlap cases. (E) Sankey diagrams illustrating the distribution of true‐positive liver cancer samples with single‐marker positivity. Samples are grouped by stage to show the contribution of each single marker.

We analysed the detection performance of the LC‐HMC model across different clinical stages of liver cancer. The LC‐HMC model showed increasing sensitivities from stage I to stage IV [93.10% (95%CI, 89.78–95.40%), 94.20% (95%CI, 86.02–97.72%), 97.35% (95%CI, 92.49–99.10%) and 100.00% (95%CI, 82.41–100.00%), respectively] (Figure 5A). Simultaneously, tests for the liver cancer‐specific protein biomarker AFP were performed in the enrolled participants. Not only the LC‐HMC model, but also the single methylation markers, RNF135, CHFR, PAX5, displayed obviously higher sensitivities compared with AFP. Furthermore, when considering the comprehensive performance across three metrics, sensitivity, specificity and accuracy, the LC‐HMC model consistently achieved the highest performance indices (Table 3). From the perspective of sensitivities across clinical stages of liver cancer, the LC‑HMC model consistently showed higher sensitivities than AFP at every stage, particularly in stage‐I (0.93 vs. 0.54) (Figure 5A). In addition, it is worth mentioning that a large proportion of liver cancer cases were at stage‐I (319/521, 61.23%), most of whom were from the diagnosing group (267 patients). In stage‐I patients from the diagnosing group, the sensitivity reached 92.9% (Figure 5B), further demonstrating the model's early detection capability. Furthermore, we analysed the diagnostic overlap between AFP and the LC‐HMC model and found that LC‐HMC detected 194 AFP‐negative cases (AFP−, LC‐HMC+), whereas AFP additionally identified 17 LC‐HMC‐negative cases (AFP+, LC‐HMC−). In stage‐I patients, these numbers were 139 and 13, respectively. Using a parallel combined strategy of the two diagnostic tools yielded 509 detected cases, corresponding to a sensitivity of 97.7% and further reducing missed liver cancers (Figure S4).

**TABLE 3:** The performance indicators of canonical biomarkers, independent methylation markers and LC‐HMC model in the clinical trial.

LC diagnostic markers	AFP	RNF135	PAX5	CHFR	LC‐HMC model
Sensitivity (%) (95% CI)	60.81 (56.54–64.92)	84.45 (81.09–87.31)	80.42 (76.80–83.60)	86.95 (83.79–89.57)	94.43 (92.12–96.09)
Specificity (%) (95% CI)	92.09 (88.99–94.37)	94.73 (92.28–96.43)	93.63 (91.00–95.53)	88.57 (85.32–91.18)	95.16 (92.78–96.78)
Accuracy (%) (95% CI)	74.29 (66.72–72.50)	89.24 (87.14–91.03)	86.58 (84.30–88.58)	87.70 (85.49–89.61)	94.77 (93.19–96.00)

Sensitivities of the LC‐HMC model and three methylation markers in 521 liver cancer cases. Specificities in 455 benign disease cases. Accuracy in sample set consisted of 521 liver cancer cases and 455 benign disease cases.

### 3.6 Perioperative surveillance

The blood methylation detection also indicated a possible application in perioperative assessment. Among the 46 patients included in the trial who underwent preoperative and postoperative testing, 41 tested positive before surgery, while all 46 patients tested negative after surgery (Figure 5C).

### 3.7 Performance of methylation markers and their complementarity in identifying liver cancer

Consistent with preclinical findings, the LC‐HMC model demonstrated superior performance compared with individual methylation markers. While RNF135 and PAX5 achieved specificities close to the LC‐HMC model [94.73% (95%CI, 92.28–96.43%) and 93.63% (95%CI, 91.00–95.53%), respectively], their sensitivities were lower [84.45% (95%CI, 81.09–87.31%) and 80.42% (95%CI, 76.80–83.60%)]. CHFR showed a sensitivity of 86.95% (95%CI, 83.79–89.57%) but lower specificity (88.57%, 95%CI, 85.32–91.18%) (Table 3). These results encouraged us to survey the complementarity of three methylation markers in identified true‐positive cases of the clinical validation cohort. Among 492 true‐positive cases, RNF135 identified 437 (five complemented), PAX5 identified 417 (seven complemented) and CHFR identified 448 (16 complemented) (Figure 5D). Figure 5E illustrates the distribution of true‐positive samples which were single positive, stratified by clinical stage. Among stage‐I samples, the majority of single‐marker complemented true positives were identified by CHFR, followed by PAX5, with fewer identified by RNF135. Similarly, when stratified by tumour differentiation, CHFR demonstrated superior complementary performance compared with the other two markers (Figure S5).

**FIGURE 6:**
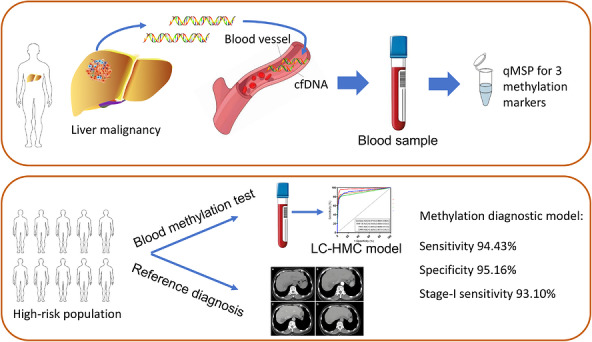
Overview of the main findings of this investigation for non‑invasive liver cancer detection, including identification of cfDNA methylation markers, model construction and validation, and its diagnostic performance.

Finally, a graphical summary presented the key findings of overall study and clinical implications of the LC‐HMC model (Figure 6).

## 4 DISCUSSION

The development of non‐invasive diagnostics has yielded a range of blood‐based biomarkers, including circulating tumour DNA (ctDNA), microRNAs, proteins, circulating tumour cells and extracellular vesicles.^19^ Among these, methylation‐based ctDNA detection has advanced rapidly and is now widely investigated not only in liver cancer but also in various other malignancies, including colorectal cancer,^20^ and multi‐cancer early detection (MCED),^21^ and so on. Beyond blood‐based tests, DNA methylation profiling in other body fluids, such as urine, has also emerged as a robust and highly accurate non‐invasive diagnostic approach.^22^, ^23^ Specifically, Chen et al. have demonstrated that urine‐based tumour DNA methylation assays for bladder cancer yield significantly higher sensitivity and specificity than traditional cytology, facilitating the effective detection of early‐stage, residual and recurrent tumours.^22^ Furthermore, recent studies have also highlighted the clinical utility of ctDNA methylation detection in cerebrospinal fluid (CSF) for the accurate diagnosis and monitoring of neurological tumours.^24^ These findings demonstrate that DNA shed by malignant cells carries distinct, tumour‐specific methylation signatures that can be reliably captured through organ‐associated body fluids, suggesting an exceptionally promising and powerful tool for early cancer diagnosis.

In this study, we identified and validated three cfDNA methylation markers, RNF135, PAX5 and CHFR, for liver cancer detection. The three‐marker combined diagnostic model is a non‐invasive approach that was not previously documented. In the research phase, using public genomic methylation datasets and in‐house experimental data, we screened 30 hypermethylated genomic sites and narrowed them to nine candidates based on liver cancer‐specific hypermethylation. Further evaluation using multiple kinds of samples identified RNF135, PAX5 and CHFR as optimal markers with excellent diagnostic stability. To integrate their diagnostic potential, we developed the LC‐HMC model using logistic regression based on training and testing sets. This logistic regression model also balanced the contribution of each marker, leveraging their unique diagnostic strengths.

In further clinical validation, a large‐scale, multi‐centre clinical trial involving 521 liver cancer patients, 455 benign disease cases and 121 other cancer cases was conducted. The LC‐HMC model achieved a sensitivity of 94.43% (95%CI, 92.12–96.09%), a specificity of 95.16% (95%CI, 92.78–96.78%) in benign diseases and 91.74% (95%CI, 85.46–95.45%) in other cancers. Notably, it maintained a sensitivity of 93.1% (95%CI, 89.78–95.40%) for stage‐I liver cancer, highlighting its potential for early detection.

The complementarity of the three methylation markers was confirmed during clinical validation, with each marker identifying true positives missed by the others. Furthermore, during the model construction phase, we analysed the Ct values of the three markers in qMSP assays. Among them, CHFR exhibited the greatest reduction in liver cancer patients compared with healthy individuals and those with benign diseases. Importantly, there was also a significant difference in CHFR methylation between healthy individuals and those with benign diseases, whereas RNF135 and PAX5 did not show such differences. This suggests that CHFR undergoes changes in its methylation pattern during the early stages of liver carcinoma in situ and the initial transition of benign disease to malignancy. This may also explain why CHFR exhibits higher sensitivity compared with the other two markers, but at the cost of slightly lower specificity. Subsequent analysis of each marker's complementarity based on clinical trial data further validated this observation. CHFR identified the highest number of single‐marker positive cases among true‐positive samples, and CHFR accounted for the majority of the stage‐I true‐positive samples with single‐marker positivity. This led us to further investigate the biological significance of markers methylation in the occurrence and progression of cancer.

CHFR is a protein involved in cell cycle regulation, specifically in delaying the onset of mitosis. Its inactivation results in uncontrolled cell proliferation, thereby promoting tumour formation.^25^ Previous studies have indicated that CHFR promoter methylation is associated with worse outcomes in stage‐II microsatellite‐stable colorectal cancer.^26^ Moreover, Li et al. reported that the promoter region of the CHFR gene exhibited high methylation status in 43% of the HCC samples, as well as in the HepG2 and SNU398 HCC cell lines.^27^ Besides, in pancreatic ductal adenocarcinoma, methylation levels at specific CpG sites within the CHFR promoter are significantly associated with patients’ progression‑free and overall survival (OS), and thus can serve as a prognostic biomarker for PDAC.^28^

RNF135 (Ring Finger Protein 135), a member of the RING finger protein family of E3 ubiquitin ligases, is primarily recognised for its role in antiviral immunity.^29^ However, its molecular mechanism in liver cancer remains unclear. A recent study by Kim et al. identified RNF135 as a distinct methylation marker for HCC that is detectable in peripheral blood.^30^ Additionally, epigenetic modifications leading to RNF135 downregulation have been shown to promote HCC cell migration. Analysis of TCGA HCC patients further demonstrated higher RNF135 methylation levels were significantly associated with poorer disease‐free survival and OS.^31^

PAX5, a key transcription factor regulating CD58 expression and B cell identity, has been implicated in several cancers. The P80R mutation in PAX5 reduces CD58 levels and contributes to blinatumomab resistance in acute lymphoblastic leukaemia.^32^, ^33^ Furthermore, a study revealed that PAX5 hypermethylation is significantly associated with poor clinical outcomes in adrenocortical carcinoma.^34^ In addition, PAX5 has been established as a tumour suppressor gene in oesophageal cancer, where promoter region methylation suppresses its expression.^35^ Consistently, our study also demonstrated that hypermethylated PAX5 serves as an indicator of liver cancer occurrence.

The biological relevance of the methylation markers to cancer supports their diagnostic utility, contributing to exceptional performance of the LC‐HMC model. This outstanding performance also stems from the rational selection of methylation markers and sample types during the discovery phase. Genomic regions with significant hypermethylation in liver cancer and minimal methylation in healthy samples were prioritised, reducing false positives. To ensure high specificity, markers exhibiting higher methylation in liver cancer compared with other cancers were selected. Peripheral blood samples, rather than tissues, were used for initial screening, focusing on cfDNA methylation applicable to blood‐based detection. Given the liver's high vascularity, cancer cells and their DNA from liver carcinoma are more likely to enter the bloodstream. Therefore, circulating DNA of liver cancer is readily detectable in blood, validating the feasibility of this approach.

To contextualise the performance of our LC‐HMC model, we considered both established clinical models and recently reported methylation‐based assays for liver cancer detection. The GALAD model, which integrates demographic and serum biomarkers (gender, age, AFP, AFP‐L3 and DCP), has demonstrated sensitivities of 81.4–91.6% and specificities of 88.2–89.7% in large validation studies.^36^, ^37^ Similarly, the GAAP model, optimised for Chinese populations using gender, age, AFP and PIVKA‐II, achieved a sensitivity of 87.2% and a specificity of 79.2%.^38^ In terms of methylation‐based methods, Xu et al. reported a 10‐methylation‐marker panel in 2017, yielding a sensitivity of 83.3% and a specificity of 90.5% in their validation cohort.^39^ The HepaAiQ model, a 20‐gene methylation panel, developed by Guo et al., achieving 86.0% sensitivity and 92.1% specificity.^40^ In 2025, other targeted assays have also emerged, including one assessing OTX1/HIST1H3G methylation (78.4% sensitivity, 93.0% specificity).^41^ Additionally, a PDTM assay introduced by Xu and colleagues evaluated the methylation of the GNB4 and Riplet in cfDNA, yielding a sensitivity of 92.3% and a specificity of 93.4%.^42^ Notably, the MCED model from the CCGA study demonstrated a sensitivity of 93.5% for liver cancer in an independent validation cohort.^20^ Compared with these approaches, the LC‐HMC model may represent a potential advancement in non‐invasive liver cancer detection, with a sensitivity of 94.43% and a specificity of 95.16%.

Despite the promising performance of these reported methods, it is crucial to benchmark our LC‐HMC model against the classic protein marker, AFP, the United States Food and Drug Administration‐approved biomarker for liver cancer.^43^ It has been reported AFP has some limitations, including a high false‐negative rate and suboptimal sensitivity (49–61%) for early‐stage disease.^44^ In the clinical trial cohort, we also assessed AFP and found that its diagnostic performance, particularly for stage‑I liver cancer, was lower than that of the LC‑HMC model. Importantly, beyond this direct comparison, we further evaluated a combined ‘either‐positive’ strategy, which demonstrated clear incremental diagnostic value over the use of AFP or the LC‐HMC model alone. Notably, the LC‐HMC model maintained high sensitivity in AFP‐negative patients (94.17% overall and 93.92% in stage‐I), effectively identifying a large proportion of cases that would otherwise be missed in current AFP‐based screening. LC‐HMC may therefore be incorporated into existing clinical pathways in several ways. Specifically, LC‐HMC could be implemented alongside AFP as a parallel blood‐based test to improve early detection or as a reflex test in AFP‐negative individuals to guide further diagnostic evaluation. In such a workflow, patients flagged by LC‐HMC could be prioritised for confirmatory imaging (e.g., contrast‐enhanced CT or MRI), thereby reducing missed diagnoses and optimising clinical decision‐making.

Remarkably, participants included in the clinical trial were enrolled in two groups, the diagnosed group and the diagnosing group, where the LC‐HMC model demonstrated excellent performance in both. The diagnosing group represents the patients who sought medical advice without prior knowledge of their definitive diagnosis. This group mainly comprised patients presenting with liver‐related or non‑specific symptoms, as well as individuals from guideline‑defined high‑risk populations for HCC. Notably, the diagnosing group included 267 patients with stage‑I liver cancer. Because early‑stage HCC is often asymptomatic or minimally symptomatic, many of these patients were enrolled primarily on the basis of their high‑risk background rather than specific alarm symptoms and were seeking medical advice or undergoing routine surveillance for underlying chronic liver disease (e.g., HBV, cirrhosis). Among the 267 stage‐I liver cancer patients in the diagnosing group, the proportion of asymptomatic high‐risk surveillance participants versus symptomatic patients was approximately 2.7:1. These inclusion methods reflect the intended use of the assay as an adjunct to ultrasound in the initial diagnostic work‐up of patients with high risk of liver cancer. Based on a sensitivity of 93.99% (95%CI, 91.28–95.90%) demonstrated in the diagnosing group, a positive result from the methylation detection would indicate a high probability of liver cancer, thereby prompting further clinical examination.

However, it should be emphasised that, although participants in the diagnosing group were prospectively enrolled, they were still recruited from a high‑risk clinical population rather than an average‑risk screening population. Thus, like the CCGA study,^20^, ^21^ our case–control study was designed to evaluate diagnostic performance in patients recruited directly from hospitals, rather than to establish effectiveness as a population‑based screening tool. In this context, the LC‑HMC model provides a cost‑effective, interpretable, PCR‑based alternative to cfDNA bisulfite‑sequencing‐based technologies for liver cancer detection and is particularly suited for initial evaluation or adjunctive diagnosis in high‑risk populations. Such an approach has the potential to complement existing liver cancer‐focused tests and broaden the toolbox of non‑invasive strategies aimed at improving outcomes in high‑risk populations. Demonstrating its performance in an average‑risk screening setting will require future large‑scale studies in broader populations, analogous to the PATHFINDER^45^ and ECLIPSE^46^ trials.

Besides, the LC‐HMC model showed high overall specificity (91.74%) for distinguishing liver cancer from other malignancies, although specificity varied across centres. This heterogeneity is likely due to differences in case composition, particularly the proportion of cancers such as gastric cancer and extrahepatic cholangiocarcinoma. These findings suggest that the observed variability reflects cohort characteristics rather than intrinsic instability of the model. Future studies with larger, more balanced and prospectively collected multi‐centre cohorts will be valuable to further confirm the model's robustness and generalisability.

While our study has demonstrated significant promise in identifying key methylation biomarkers for the early detection of liver cancer, several limitations need to be acknowledged. First, the study did not account for potential variations in cfDNA methylation patterns across different ethnic groups, which may limit the model's applicability and effectiveness in non‐Asian populations. Looking ahead, future research should prioritise international collaborative studies and include more diverse ethnic cohorts to validate the robustness of our findings across broader populations, thereby expanding its applicability and impact globally. Second, as this is a cross‐sectional study, we only present pre‐ and postoperative methylation results, which demonstrate treatment‐related signal conversion and support the malignant tumour specificity of these markers. However, in the absence of longitudinal follow‐up or recurrence sampling, these findings do not provide evidence that this assay can detect minimal residual disease (MRD). Future dedicated prospective studies with longitudinal designs, incorporating serial methylation measurements at multiple time points before and after treatments, during the treatment course, and at or around the time of recurrence, are needed to clarify how these methylation patterns evolve over time and to determine whether these markers can be reliably used for MRD detection and disease monitoring.

## 5 CONCLUSIONS

The study identified three distinguished methylation markers and built the LC‐HMC model, which serves as a non‐invasive method for diagnosing liver cancer. The large‐scale, multi‐centre, prospective clinical trial confirmed its outstanding performance in a series of clinical evaluation perspectives, indicating non‐invasive, accurate and early detection strategies in liver cancer. These results pave the way for enhancing current liver cancer screening practices and facilitating earlier intervention, potentially improving patient outcomes and presenting an advancement in the field of oncology diagnostics.

## AUTHOR CONTRIBUTIONS

Ruohan Zhang, Xin‐Rong Yang, Guangming Li, Yinan Deng, Ji‐Bing Liu, Hongjun Gao and Jie Zhao are co‐first authors of the article. These co‐first authors took responsibility for the integrity of the data and the accuracy of the figures and tables presented in the study, as well as the manuscript drafting. Hua Li, Yonghong Zhang, Xiaoliang Han, Jia Fan, Jian Zhou, Kefeng Dou and Kaishan Tao were responsible for the study conception and design. Jianwen Cheng, Xiaofei Zhao, Yang Yang, Zhen Wu, Shuangzhen Gu, Yang Wu, Zhongying Ma, Yanli Liu, Yan Kang and Guangpeng Zhou conducted the experiments. Ruohan Zhang, Xin‐Rong Yang, Guangming Li, Yinan Deng, Ji‐Bing Liu and Hongjun Gao were responsible for managing patients and data acquisition. Jie Zhao, Xiaoliang Han and Kaishan Tao took responsibility for the interpretation of the data. Hua Li, Yonghong Zhang, Xiaoliang Han, Jia Fan, Jian Zhou, Kefeng Dou and Kaishan Tao checked and confirmed the conclusion and the final manuscript.

## CONFLICT OF INTEREST STATEMENT

The authors declare the following potential conflict of interest with respect to the research, authorship and/or publication of this article: Dr. Jie Zhao, Zhen Wu, Shuangzhen Gu, Yang Wu, Dr. Guangpeng Zhou and Dr. Xiaoliang Han are current employees of BioChain (Beijing) Science & Technology, Inc. The other authors declare no conflicts of interest.

## ETHICS STATEMENT

All the procedures of the study were conducted in accordance with the Helsinki Declaration of 1964 and later versions. All the participants in the study provided written informed consent and were duly informed about the utilisation of plasma and test outcomes. The study was approved by the local ethics committees of each hospital involved. The ethical numbers are as follows: *Centres participated in preclinical investigation*: Cancer Hospital of Shandong First Medical University, SDTHEC 2023011011; The First Affiliated Hospital of Fourth Military Medical University, Xijing Hospital, QX20231022‐1; Shanxi Cancer hospital, KY2023103. *Centres participated in clinical trial*: The First Affiliated Hospital of Fourth Military Medical University, Xijing Hospital, QX20231022‐C‐1; Zhongshan Hospital, Fudan University, 2023–077; Beijing Youan Hospital, Capital Medical University, LL‐2023‐075‐S; The Third Affiliated Hospital, Sun Yat‐sen University, SJ2023‐004‐01.

## CONSENT

All authors of this article consent to its publication.

## Supporting information



Supporting Information

Supporting Information

## Data Availability

The data of TCGA cohort were downloaded from the GDC data Portal website (https://portal.gdc.cancer.gov/). The original data and the clinical information of the clinical trial cohort are displayed in Supporting Information. The other data analysed in this study are available upon reasonable request from the corresponding author.
