# Supplementary material for: A multi‐centre, prospective trial of a methylation‐based liquid biopsy for early detection of liver cancer in high‐risk populations
Source: Clin Transl Med. 2026 May 18;16(5):e70687. doi: 10.1002/ctm2.70687 (PMC13181335; doi:10.1002/ctm2.70687)
Supplement: Supplementary file 1 — Supporting Information [file CTM2-16-e70687-s001.docx]

**A Multicenter, Prospective Trial of a Methylation-Based Liquid Biopsy for Early Detection of Liver Cancer in High-Risk Populations**

**Supplementary materials**

Ruohan Zhang^1,#^, Xinrong Yang^2,#^, Guangming Li^3,7,#^, Yinan Deng^4,#^, Jibing Liu^5,#^, Hongjun Gao^6,10,#^, Jie Zhao^12,#^, Jianwen Cheng^2^, Xiaofei Zhao^3,7^, Yang Yang^4^, Zhen Wu^12^, Shuangzhen Gu^12^, Yang Wu^12^, Zhongying Ma^11^, Yanli Liu^9^, Yan Kang^10^, Guangpeng Zhou^12^, Hua Li^4*^, Yonghong Zhang^7,8,*^, Xiaoliang Han^12,*^, Jia Fan^2,*^, Jian Zhou^2,*^, Kefeng Dou^1,*^, Kaishan Tao^1,*^

^1^ Department of Hepatobiliary Surgery, Xijing Hospital, Air Force Medical University, Xi’an, 710032, China

^2^ Department of Liver Surgery & Transplantation, Liver Cancer Institute, Zhongshan Hospital, Fudan University, and Key Laboratory of Carcinogenesis and Cancer Invasion, Ministry of Education, Shanghai, 200032, China

^3^ Department of General Surgery Center, Beijing Youan Hospital, Capital Medical University, Beijing, 100069, China

^4^ Department of Hepatic Surgery & Liver Transplantation, The Third Affiliated Hospital of Sun Yat-sen University, Guangzhou, 510630, China

^5^ Department of Interventional Therapy Ⅰ, Shandong Cancer Hospital and Institute, Shandong First Medical University and Shandong Academy of Medical Sciences, Jinan, Shandong, 250117, China.

^6^ Department of Clinical Laboratory, State Key Laboratory of Molecular Oncology, National Cancer Center/National Clinical Research Center for Cancer/Cancer Hospital, Chinese Academy of Medical Sciences and Peking Union Medical College, Chaoyang District, Beijing, China

^7^ Clinical Center for Liver Cancer, Capital Medical University, Beijing, 100069, China

^8^ Interventional therapy Center for liver diseases and cancer, Beijing Youan Hospital, Capital Medical University, Beijing, 100069, China

^9^ Shandong Cancer Hospital and Institute, Shandong First Medical University and Shandong Academy of Medical Sciences, Shandong Provincial Key Laboratory of Precision Oncology, Jinan, Shandong, 250117, China

^10^ Department of Clinical Laboratory, Shanxi Province Cancer Hospital, Shanxi Hospital Chinese Academy of Medical Sciences, Taiyuan, Shanxi, China

^11^ Department of Pharmacy, Xijing Hospital, Air Force Medical University

^12^ BioChain (Beijing) Science & Technology Inc., Beijing 102600, China

# These authors contributed equally to this work.

* Correspondence authors:

Kaishan Tao: [taokaishan0686@163.com](mailto:taokaishan0686@163.com); Tel: 13909250686

Kefeng Dou: [doukef@fmmu.edu.cn;](mailto:doukef@fmmu.edu.cn;) Tel: 13571890758

Jian Zhou: [zhou.jian@zs-hospital.sh.cn](mailto:zhou.jian@zs-hospital.sh.cn);

Jia Fan: [fan.jia@zs-hospital.sh.cn](mailto:fan.jia@zs-hospital.sh.cn);

Xiaoliang Han: [sean.han@biochainbj.com;](mailto:shan@biochain.com;) Tel: 18612824530

Yonghong Zhang: [13810108505@163.com](mailto:13810108505@163.com); Tel: 13810108505

Hua Li: [lihua3@mail.sysu.edu.cn](mailto:lihua3@mail.sysu.edu.cn); Tel: 13060975202

**Content**

1. Supplementary methods

2. Supplementary figures and Supplementary figure legends

Supplementary Figure S1

Supplementary Figure S2

Supplementary Figure S3

Supplementary Figure S4

3. Supplementary Table

Supplementary Table S1

Supplementary Table S2

Supplementary Table S3

Supplementary Table S4

Supplementary Table S5

Supplementary Table S6

Supplementary Table S7

**1. Supplementary methods**

**Chip-450k data collection and analysis for the TCGA LIHC cohort**

The DNA methylation public data of the TCGA LIHC cohort were downloaded from the data portal (https://portal.gdc.cancer.gov/projects/TCGA-LIHC) for 377 liver hepatocellular carcinoma cases, including 377 liver cancer tissues and 50 matched adjacent non-cancerous tissues. We performed differential methylation analysis using the R package ChAMP ^[17]^ (with the BMIQ method for batch effect correction and the Benjamini-Hochberg method for p-value adjustment) on Chip-450k data of 377 liver cancer tissues and 50 matched adjacent non-cancerous tissues derived from TCGA dataset. We obtained sites with p-values <0.01 and selected those with higher methylation levels in cancer tissues compared to normal samples (delta_T2N >0.29), resulting in 901 cancer-hypermethylated genomic sites.

**Panel-targeted bisulfite sequencing**

Based on the obtained 901 hypermethylation genomic sites and the collected hotspot genes associated with liver cancer, we designed a methylation detection panel of 28.7 Mb covering 3,311,582 CpGs. We conducted panel-targeted methylation sequencing on 559 clinical blood samples collected during the biomarker discovery phase. First, we extracted cfDNA from 3.5 mL of plasma using the QIAamp Circulating Nucleic Acid Kit (Cat. No.55114, QIAGEN). Next, cfDNA was bisulfite-converted using the EZ DNA Methylation-Gold Kits (D5005, ZYMO RESEARCH), resulting in bisulfite-converted DNA for library preparation. The xGen™ Methyl-Seq DNA Library Prep Kit (10009824, IDT) was used to prepare libraries from the conversion products, which were then hybrid-captured using a custom probe panel from TWIST. Libraries passing quality control were sequenced using the Illumina NovaSeq with the S4 standard workflow, achieving an average sequencing depth of >150X.

**Panel-targeted sequencing data analysis**

We used fastp to conduct quality control and filtering of raw panel-targeted sequencing data ^[15]^. Cleaned sequencing reads were then aligned to the human genome (version: Hg19) using Bismark, with PCR duplicates removed. Subsequently, Bismark was used to analyze the CpG methylation levels detected in samples to provide methylation profiles for target regions ^[16]^. Differentially methylated sites were selected based on criteria: 1) methylation levels in healthy individuals <0.015 as calculated by Bismark, and 2) median and average methylation levels in the healthy cohort being lower than those in the liver cancer cohort. After filtering, the top 30 genes by methylation level were selected for further study, and 9 candidate methylation markers were identified.

**Optimal methylation markers screening**

Primers and probes for quantitative methylation-specific PCR (qMSP) were designed for the 9 candidate methylation markers. These were tested across 32 liver cancer tissue samples, 24 liver cancer cell lines, 32 healthy plasma samples, and 9 liver cancer plasma samples, with sensitivity/specificity statistics compiled for different sample types. Scores for each candidate marker were calculated based on the number of samples, using the formula:

*Score = 10×tissue sensitivity + 7.5×cell line sensitivity + 10×healthy plasma specificity + 2.8125×cancer plasma sensitivity*

After comprehensive consideration of their scores and true positive samples they covered, the optimal 3 methylation markers were selected.

**Model construction**

The three selected methylation markers were tested using qMSP experiments on clinical blood samples. Logistic regression models were built based on the methylation data obtained. First, results from 280 clinical plasma samples were used as training set, with analyzing ROC curves for a three-gene combined diagnostic model and individual genes. Subsequently, this study used data from 124 clinical plasma samples as the testing set, with analyzing ROC curves for the combined model and individual genes.

**Sample collection and storage**

Blood samples from clinical participants were collected and recorded. 5 mL of peripheral blood was drawn using 6 mL K2EDTA anticoagulant tubes (BD Vacutainer®), with plasma collected within 8 hours of blood draw. After collection, blood samples were centrifuged at 1350±150 rcf (Relative Centrifugal Force) for 12 minutes, then transferred to 15 mL centrifuge tubes. The procedure was repeated. Prepared plasma samples were immediately stored at (-25 ~ -15)°C and analyzed within two weeks.

**DNA isolation and bisulfite conversion**

The experiment was conducted using a Nucleic Acid Extraction Kit (BioChain (Beijing) Science & Technology, Inc., China). There was 2.1 mL of plasma mixed with 2.1 mL of lysis buffer, and the mixture was left to stand for 10 minutes. Then, 60 µL of magnetic beads and 1.5 mL of absolute ethanol were added and mixed at room temperature for 45 minutes, after which the supernatant was discarded using magnetic separation. After adding 1.5 mL of Wash Solution A and transferring to a 2.0 mL centrifuge tube, the supernatant was discarded using magnetic separation, following by adding 100 µL of elution buffer and mixing at 80°C for10 minutes. The cfDNA concentrations ranged from approximately 2–50 ng/mL of plasma, with a mean concentration of about 9 ng/mL. After extraction, at least 10 ng of cfDNA per sample was used for subsequent bisulfite conversion and methylation assays. The fragment size distribution of qualified cfDNA showed a characteristic mononucleosomal peak at approximately 170 bp, with the majority of fragments within the 150–250 bp range and no substantial high–molecular weight genomic DNA contamination.

The cfDNA was transferred to a 2.0 mL centrifuge tube, where 150 µL of bisulfite solution and 25 µL of protection buffer were added. The solution was incubated at 80°C for 45 minutes. Following washing by solution A and B, and drying steps, 37 µL of elution buffer was added. And the bisulfite-converted DNA sample was thoroughly mixed, ready for PCR amplification. The bisulfite conversion efficiency was calculated to be 99.58%, indicating highly efficient conversion.

**Quantitative methylation specific PCR (qMSP)**

The bisulfite-converted DNA was used as a template for PCR amplification in a total volume of 50 µL, comprising 25 µL of a prepared reaction mix and 25 µL of bisulfite-converted DNA. The amplification protocol was as follows: 94°C for 20 minutes, followed by 45 cycles of 57°C for 35 seconds and 93°C for 30 seconds. An Applied Biosystems7500 Fast Real-Time PCR System or SLAN-96S automatic medical PCR analysis system (Shanghai Hongshi Medical Technology Co., Ltd.) was used. Each assay system included a patient sample, and positive and negative quality controls. PCR detection targeted methylated RNF135, PAX5, and CHFR DNA, with ACTB DNA serving as house-keeping gene control. For each patient, one cfDNA sample was extracted from peripheral blood, and a single qMSP reaction was performed without technical replicates (duplicate holes were not set).

**2. Supplementary figures and Supplementary figure legends**


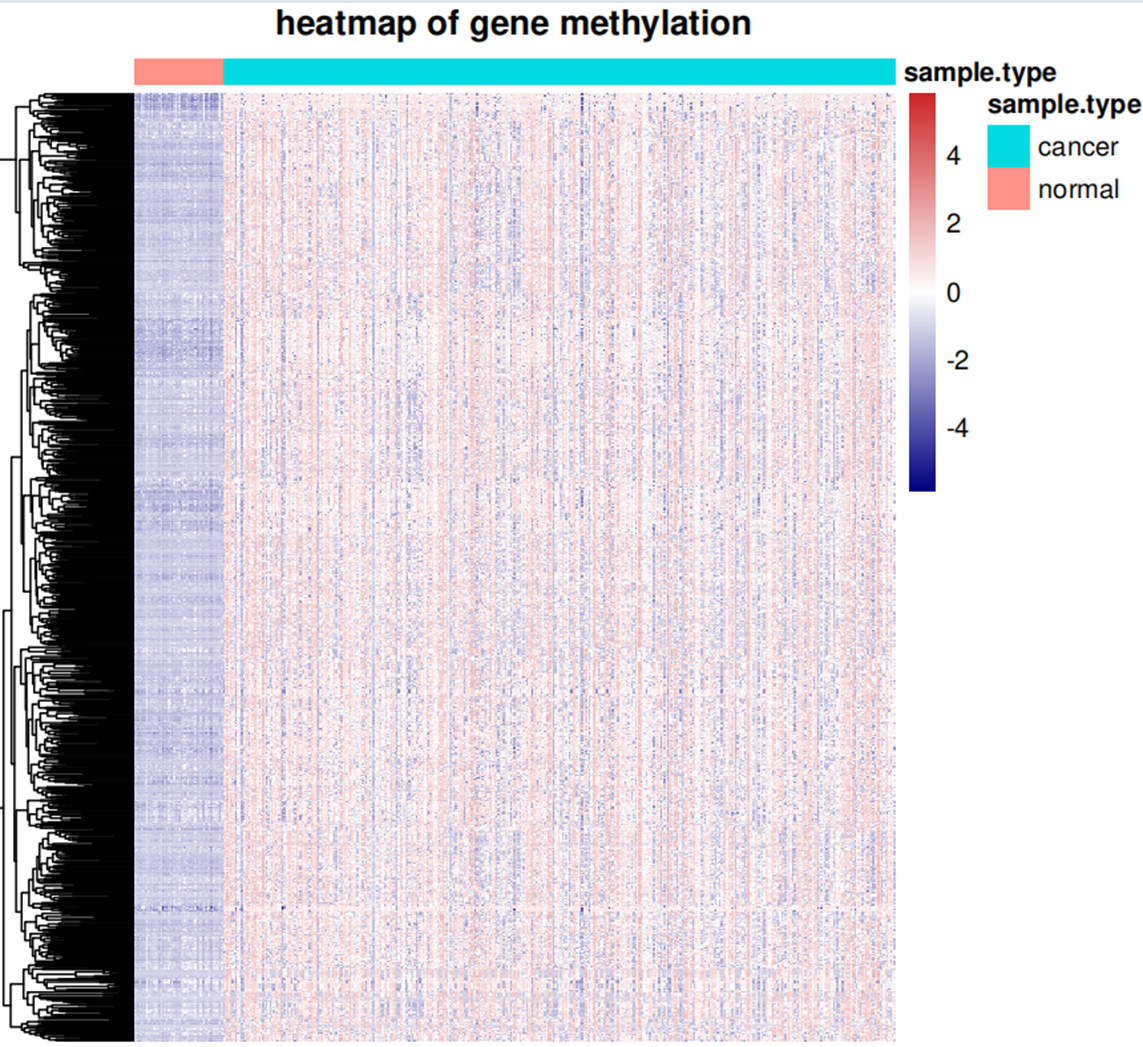


**Supplementary Figure S1.** **Abundant numbers of differential methylation regions were hypermethylated in liver cancer tissues.** Differential methylation analysis based on DNA methylation 450K array data from the TCGA LIHC (Liver hepatocellular carcinoma) cohort, downloaded from the data portal (https://portal.gdc.cancer.gov/projects/TCGA-LIHC), 377 liver cancer tissues vs. 50 adjacent normal tissues.


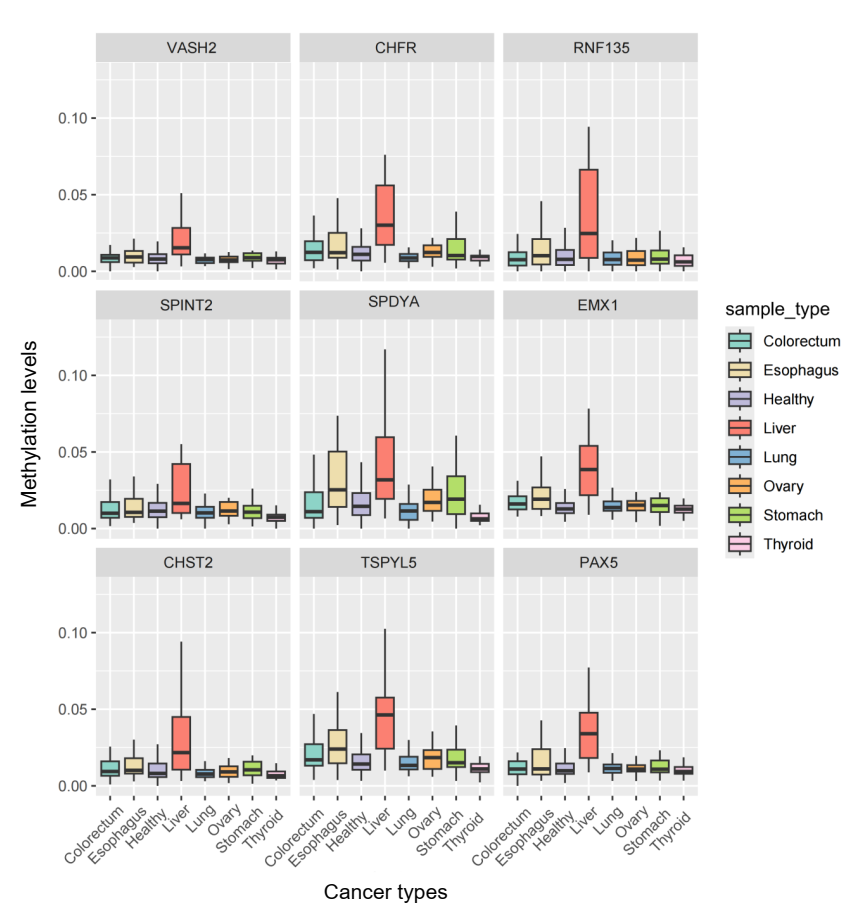


**Supplementary Figure S2. Screening methylation markers through conducting panel-targeted sequencing on in-house clinical blood samples.** Box plots indicate the methylation levels of 9 genes in blood samples from 67 cases of colorectal cancer, 30 cases of esophageal cancer, 31 cases of liver cancer, 51 cases of lung cancer, 37 cases of ovarian cancer, 46 cases of gastric cancer, 26 cases of thyroid cancer and 178 healthy individuals. The samples were collected from two centers participated in preclinical investigation. The box plot illustrates the interquartile range (IQR), with the line within the box denoting the median of the data.


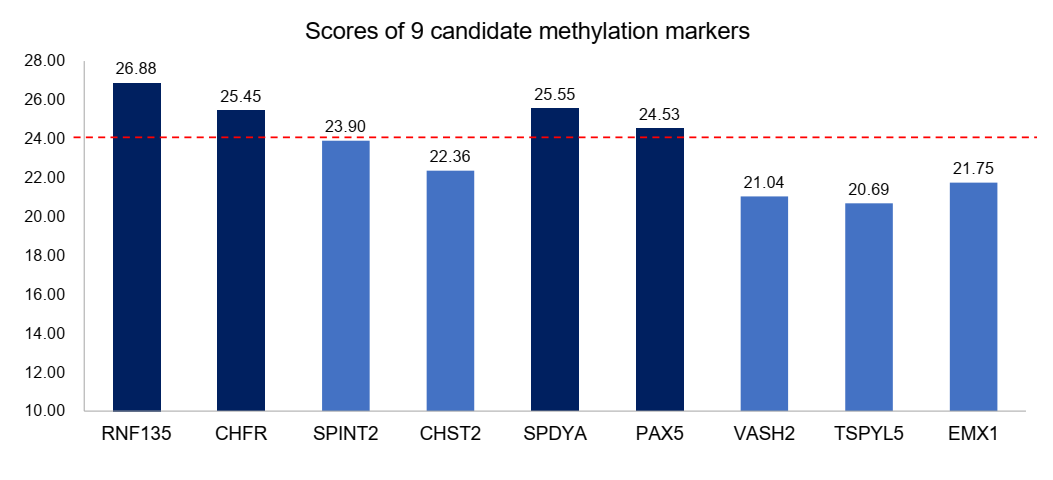


**Supplementary Figure S3. Evaluating 9 candidate methylation markers by conducting qMSP on multiple kinds of samples and scoring each of markers based on their performance in each type of samples.** Bar plot indicating the scores of each candidate markers calculated through the formula: Score=10*tissue sensitivity+7.5*cell line sensitivity+10*healthy plasma specificity+2.8125*cancer plasma sensitivity.


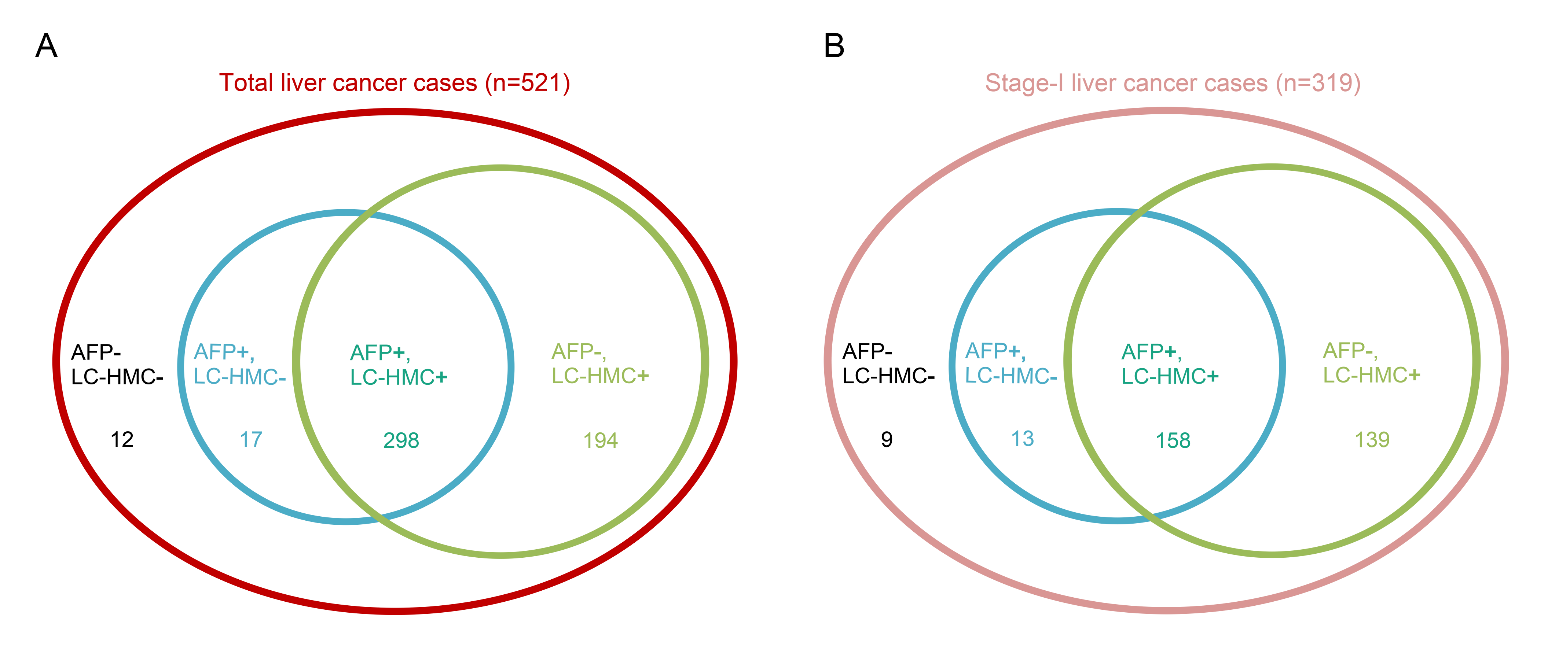


**Supplementary Figure S4. Diagnostic overlap between the LC-HMC model and AFP in liver cancer detection. (A)** Diagram illustrating the distribution of positive and negative detection by the LC-HMC model and AFP across the overall 521 liver cancer cases. **(B)** Diagram showing the distribution of positive and negative detection by the LC-HMC model and AFP across the early-stage (Stage-I) liver cancer cases.


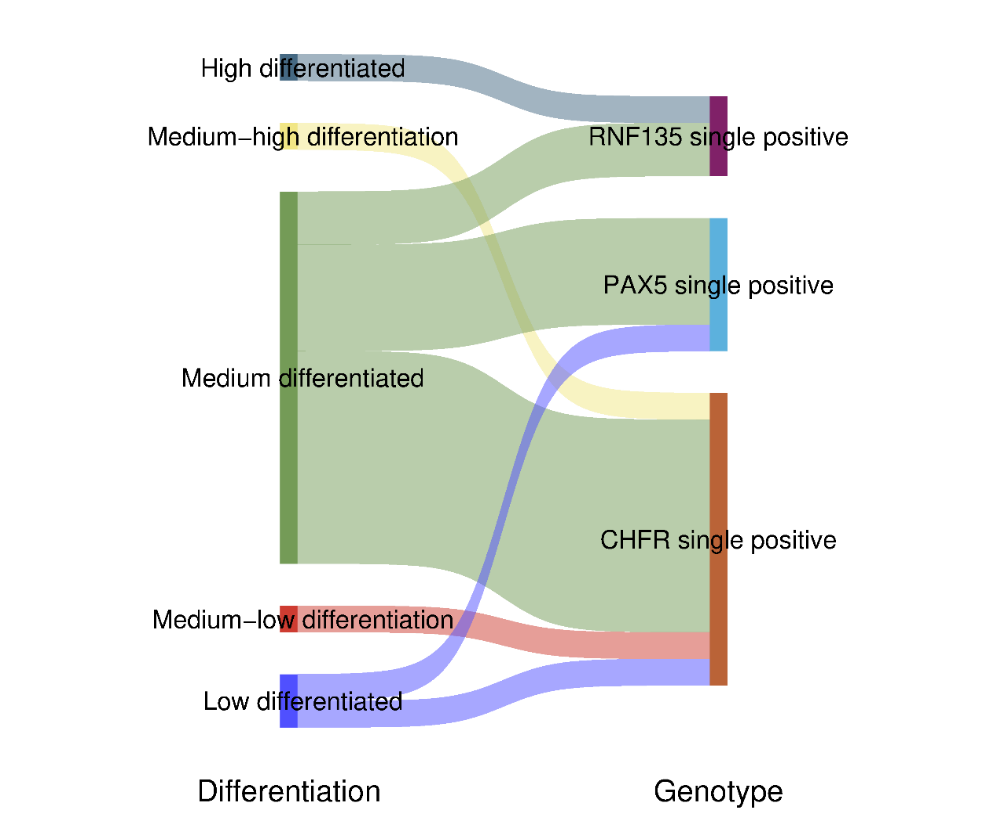


**Supplementary Figure S5.** Sankey diagrams illustrating the distribution of true positive liver cancer samples with single-marker positivity. Samples are grouped by tumor differentiation to show the contribution of each single marker.

**3. Supplementary Tables**

**Supplementary Table S1.** The cutoff values of automated chemiluminescence kits used in different centers.

| **Center** | **AFP (ng/ml)** |
| --- | --- |
| The First Affiliated Hospital of Fourth Military Medical University, Xijing Hospital (Xi’an, China) | 7.0 |
| Zhongshan Hospital Fudan University (Shanghai, China) | 8.0 |
| Beijing Youan Hospital, Capital Medical University (Beijing, China) | 7.0 |
| The Third Affiliated Hospital, Sun Yat-sen University (Guangzhou, China) | 8.1 |

**Supplementary Table S2.** Clinical characteristics of the model-construction sets in preclinical investigation.

| **Features** | **Training set** | | **Test Set** | |
| --- | --- | --- | --- | --- |
|  | Liver cancer | Control | Liver cancer | Control |
| Sample volume | 91 | 189 | 48 | 76 |
| Age (years), Mean±SD | 56±10.63 | 59±11.36 | 59 ±8.88 | 57±12.24 |
| Gender, n (%) |  |  |  |  |
| Male | 58 (63.74%) | 96 (50.79%) | 34 (70.93%) | 35 (46.05%) |
| Female | 33 (36.26%) | 91 (48.15%) | 14 (29.17%) | 41 (53.95%) |
| Pathologic stage (AJCC 8th), n |  |  |  |  |
| I | 34 |  | 16 |  |
| II | 24 |  | 12 |  |
| III | 24 |  | 9 |  |
| IV | 7 |  | 8 |  |
| Unknown | 2 |  | 3 |  |

**Supplementary Table S3.** Clinical characteristics of the clinical trial cohort.

| **Variables** | **Liver cancer** | **Benign disease control** | **Other cancers** |
| --- | --- | --- | --- |
| Sample volume | 521 | 455 | 121 |
| Age (years), Mean±SD | 57±10.94 | 48±12.72 | 62±10.46 |
| Gender, n (%) |  |  |  |
| Male | 443 (85.03%) | 243 (53.41%) | 75 (61.98%) |
| Female | 78 (14.97%) | 212 (46.59%) | 46 (38.02%) |
| Pathologic stage (AJCC 8th), n (%) |  |  |  |
| I | 319 (61.23%) |  |  |
| II | 69 (13.24%) |  |  |
| III | 113 (21.69%) |  |  |
| IV | 18 (3.45%) |  |  |
| Unknown | 2 (0.38%) |  |  |
| Liver cancer subtypes, n (%) |  |  |  |
| Hepatocellular carcinoma | 500 (95.97%) |  |  |
| Mixed hepatocellular-cholangiocarcinoma | 21 (4.03%) |  |  |
| Differentiation, n (%) |  |  |  |
| Low differentiation | 35 (6.72%) |  |  |
| Medium-low differentiation | 38 (7.29%) |  |  |
| Medium differentiation | 228 (43.76%) |  |  |
| Medium-high differentiation | 11 (2.11%) |  |  |
| High differentiation | 14 (2.69%) |  |  |
| Unknown | 195 (37.43%) |  |  |
| Control liver lesions, n (%) |  |  |  |
| Liver cirrhosis |  | 97 (21.10%) |  |
| Liver benign tumor |  | 224 (49.23%) |  |
| Hepatitis B |  | 72 (15.82%) |  |
| Hepatitis C |  | 32 (7.03%) |  |
| Other hepatitis |  | 4 (1.10%) |  |
| Fatty liver |  | 26 (5.71%) |  |
| Other cancer types, n (%) |  |  |  |
| Extrahepatic cholangiocarcinoma |  |  | 15 (12.40%) |
| Pancreatic cancer |  |  | 21 (17.36%) |
| Esophageal cancer |  |  | 24 (19.83%) |
| gastric cancer |  |  | 30 (24.79%) |
| Colon cancer |  |  | 31 (25.62%) |

**Supplementary Table S4.** Clinical characteristics of the diagnosed and diagnosing group in clinical trial cohort.

|  | Diagnosed group | | | Diagnosing group | | |
| --- | --- | --- | --- | --- | --- | --- |
|  | Liver cancer | Benign disease control | Other cancer | Liver cancer | Benign disease control | Other cancer |
| Case number, n | 105 | 232 | 114 | 416 | 223 | 7 |
| Age, years, mean±SD | 58.67±10.28 | 50.06±13.39 | 62.45±9.81 | 57.26±11.34 | 48.05±12.46 | 63.29±6.68 |
| Gender, n (%) |  |  |  |  |  |  |
| Male | 93 (88.57%) | 124 (53.45%) | 70 (61.40%) | 350 (84.13%) | 119 (53.36%) | 5 (71.43%) |
| Female | 12 (11.43%) | 108 (46.55%) | 44 (38.60%) | 66 (15.87%) | 104 (46.64%) | 2 (28.57%) |
| Stage (AJCC 8th), n (%) |  |  |  |  |  |  |
| I | 52 (49.52%) |  |  | 267 (64.18%) |  |  |
| II | 18 (17.14%) |  |  | 51 (12.26%) |  |  |
| III | 30 (28.57%) |  |  | 83 (19.95%) |  |  |
| IV | 4 (3.81%) |  |  | 14 (3.37%) |  |  |
| Unknown | 1 (0.95%) |  |  | 1 (0.24%) |  |  |
| Liver cancer subtypes, n (%) |  |  |  |  |  |  |
| Hepatocellular carcinoma | 103 (98.10%) |  |  | 397 (95.43%) |  |  |
| Mixed hepatocellular-cholangiocarcinoma | 2 (1.90%) |  |  | 19 (4.57%) |  |  |
| Control liver lesions, n (%) |  |  |  |  |  |  |
| Liver cirrhosis |  | 49 (21.12%) |  |  | 48 (21.52%) |  |
| Liver benign tumor |  | 142 (61.21%) |  |  | 82 (36.77%) |  |
| Hepatitis B |  | 24 (10.34%) |  |  | 48 (21.52%) |  |
| Hepatitis C |  | 7 (3.02%) |  |  | 25 (11.21%) |  |
| Other hepatitis |  | 1 (0.43%) |  |  | 3 (1.35%) |  |
| Fatty liver |  | 9 (3.88%) |  |  | 17 (7.62%) |  |
| Other cancer types, n (%) |  |  |  |  |  |  |
| Extrahepatic cholangiocarcinoma |  |  | 10 (8.77%) |  |  | 5 (71.43%) |
| Pancreatic cancer |  |  | 19 (16.67%) |  |  | 2 (28.57%) |
| Esophageal cancer |  |  | 24 (21.05%) |  |  | 0 (0.00%) |
| gastric cancer |  |  | 30 (26.32%) |  |  | 0 (0.00%) |
| Colon cancer |  |  | 31 (27.19%) |  |  | 0 (0.00%) |

**Supplementary Table S5.** The performance of the LC-HMC model in the clinical trial.

| **Indicators** | **Overall** | **Diagnosed group** | **Diagnosing group** |
| --- | --- | --- | --- |
| Sensitivity (%) (95% CI) | 94.43 (92.12~96.09) | 96.19 (90.61~98.51) | 93.99 (91.28~95.90) |
| Specificity (%) (95% CI) | 95.16 (92.78~96.78) | 94.83 (91.18~97.02) | 95.52 (91.95~97.55) |
| Accuracy (%) (95% CI) | 94.77 (93.19~96.00) | 95.25 (92.43~97.06) | 94.52 (92.47~96.03) |

**Supplementary Table S6.** The performance of LC-HMC model in 4 centers participated in clinical trial.

|  | **Liver cancer** | **Benign disease control** | **Other cancer** |
| --- | --- | --- | --- |
| **Xijing Hospital** |  |  |  |
| Case number | 108 | 170 | 43 |
| Test-positive cases | 103 | 6 | 1 |
| Test-negative cases | 5 | 164 | 42 |
| Sensitivity for LC patients (95%CI) | 95.37%  (89.62%~98.01%) |  |  |
| Specificity for benign disease control (95%CI) |  | 96.47%  (92.51%~98.37%) |  |
| Specificity for other cancers (95%CI) |  |  | 97.67%  (87.93%~99.59%) |
| **Zhongshan Hospital** |  |  |  |
| Case number | 130 | 103 | 65 |
| Test-positive cases | 121 | 6 | 7 |
| Test-negative cases | 9 | 97 | 58 |
| Sensitivity for LC patients (95%CI) | 93.08%  (87.37%~96.32%) |  |  |
| Specificity for benign disease control (95%CI) |  | 94.17%  (87.86%~97.30%) |  |
| Specificity for other cancers (95%CI) |  |  | 89.23%  (79.40%~94.68%) |
| **Beijing Youan Hospital** |  |  |  |
| Case number | 195 | 105 | 9 |
| Test-positive cases | 183 | 7 | 2 |
| Test-negative cases | 12 | 98 | 7 |
| Sensitivity for LC patients (95%CI) | 93.85%  (89.56%~96.45%) |  |  |
| Specificity for benign disease control (95%CI) |  | 93.33%  (86.87%~96.73%) |  |
| Specificity for other cancers (95%CI) |  |  | 77.78%  (45.26%~93.68%) |
| **The Third Affiliated Hospital, Sun Yat-sen University** |  |  |  |
| Case number | 88 | 77 | 4 |
| Test-positive cases | 85 | 3 | 0 |
| Test-negative cases | 3 | 74 | 4 |
| Sensitivity for LC patients (95%CI) | 96.59%  (90.45%~98.83%) |  |  |
| Specificity for benign disease control (95%CI) |  | 96.10%  (89.15%~98.66%) |  |
| Specificity for other cancers (95%CI) |  |  | 100.00%  (51.01%~100.00%) |

**Supplementary Table S7.** The performance in LC-HMC model applied in male and female.

|  | Male | | | Female | | |
| --- | --- | --- | --- | --- | --- | --- |
|  | Liver cancer | Benign disease control | Other cancers | Liver cancer | Benign disease control | Other cancers |
| Case number | 443 | 243 | 75 | 78 | 212 | 46 |
| Test-positive cases | 416 |  |  | 76 |  |  |
| Test-negative cases |  | 230 | 70 |  | 203 | 41 |
| Sensitivity for LC patients (95%CI) | 93.91%  (90.68% ~ 95.42%) |  |  | 97.44%  (89.72%~99.22%) |  |  |
| Specificity for benign disease control (95%CI) |  | 94.65%  (89.47%~94.42%) |  |  | 95.75%  (89.78%~97.22%) |  |
| Specificity for other cancers (95%CI) |  |  | 93.33%  (84.22%~97.27%) |  |  | 89.13%  (77.89%~96.27%) |

**Reference**

15 Chen, S., Zhou, Y., Chen, Y. & Gu, J. fastp: an ultra-fast all-in-one FASTQ preprocessor. Bioinformatics (Oxford, England) 34, i884-i890, doi:10.1093/bioinformatics/bty560 (2018).

16 Krueger, F. & Andrews, S. R. Bismark: a flexible aligner and methylation caller for Bisulfite-Seq applications. Bioinformatics (Oxford, England) 27, 1571-1572, doi:10.1093/bioinformatics/btr167 (2011).

17 Morris, T. J. et al. ChAMP: 450k Chip Analysis Methylation Pipeline. Bioinformatics (Oxford, England) 30, 428-430, doi:10.1093/bioinformatics/btt684 (2014).
